# Insights into the timing, intensity and natural setting of Neanderthal occupation from the geoarchaeological study of combustion structures: A micromorphological and biomarker investigation of El Salt, unit Xb, Alcoy, Spain

**DOI:** 10.1371/journal.pone.0214955

**Published:** 2019-04-24

**Authors:** Lucia Leierer, Margarita Jambrina-Enríquez, Antonio V. Herrera-Herrera, Rory Connolly, Cristo M. Hernández, Bertila Galván, Carolina Mallol

**Affiliations:** 1 Instituto Universitario de Biorgánica Antonio González, Universidad de La Laguna, La Laguna, Santa Cruz de Tenerife, Spain; 2 Departamento de Geografía e Historia, Área de Prehistoria (Facultad de Humanidades), Universidad de La Laguna, Campus de Guajara, La Laguna, Santa Cruz de Tenerife, Spain; Max Planck Institute for the Science of Human History, GERMANY

## Abstract

Middle Paleolithic lithic and faunal assemblages throughout Eurasia reflect short-term Neanderthal occupations, which suggest high group mobility. However, the timing of these short-term occupations, a key factor to assess group mobility and territorial range, remains unresolved. Anthropogenic combustion structures are prominent in the Middle Paleolithic record and conceal information on the timing and intensity and natural setting of their associated human occupations. This paper examines a concentration of eleven combustion structures from unit Xb of El Salt, a Middle Paleolithic site in Spain through a geoarchaeological approach, in search of temporal, human impact and paleoenvironmental indicators to assess the timing, intensity and natural setting of the associated human occupations. The study was conducted using micromorphology, lipid biomarker analysis and compound specific isotope analysis. Results show in situ hearths built on different diachronic topsoils rich in herbivore excrements and angiosperm plant residues with rare anthropogenic remains. These data are suggestive of low impact, short-term human occupations separated by relatively long periods of time, with possible indicators of seasonality. Results also show an absence of conifer biomarkers in the mentioned topsoils and presence of conifer charcoal among the fuel residues (ash), indicating that fire wood was brought to the site from elsewhere. A microscopic and molecular approach in the study of combustion structures allows us to narrow down the timescale of archaeological analysis and contributes valuable information towards an understanding of Neanderthal group mobility and settlement patterns.

## 1 Introduction

What is the approximate amount of time that Neanderthal groups occupied any given place in the landscape? How much time passed before they returned to that same place? These questions are key to understand Neanderthal group mobility and settlement patterns.

So far, these questions have not been properly answered using the current proxies used in Middle Paleolithic archaeology.

Some proxies are especially helpful for distinguishing broadly between long-term and short-term occupations. Cleaning behaviors and maintenance activities can point to long-term occupations [1–5] as evidenced by raked-out hearths [6–9] or thick ash deposits indicative of recurrent refueling [10–12]. The origin of lithic raw material might help in differentiating long-term occupation (raw material acquisition nearby the site) from short-term occupations (raw material acquisition from further away as reflective of high group mobility) [1,13]. Additionally, occupation length estimates can be made on the basis of material assemblage accumulation density [14,15]. Also, carnivore activity on the faunal assemblages might indicate short term occupations, since leftover bones would still be fresh and more appealing to carnivores [1,16]. Another method that has been used to frame the timing of human occupations in archaeological context is dental wear analysis which looks at seasonal markers in anthropogenic tooth remains to identify the season in which the animal has died. A stratigraphic unit with several teeth representing the same season commonly imply different, diachronic short-term seasonal occupations [17,18].

So far, based on a combination of the above-mentioned proxies, the Middle Paleolithic archaeological record throughout Europe and the Near East, reflects short-term human occupations [14,15,18–33].

Still, the timing of Neanderthal short-term occupations remains unresolved. A key to address this issue is to isolate single occupation episodes. Most proxies of occupation duration are based on the analysis of remains recovered from archaeological palimpsest deposits [34–38] representing several occupation episodes. Dissecting Middle Paleolithic palimpsest deposits into single occupation episodes is not only essential to approach human behavior and change [26,39,40], but is also a prerequisite to assess the time between human occupations. In Paleolithic contexts archaeological palimpsests can be dissected through lithic raw material units, faunal and lithic refits, archaeostratigraphy or tooth microwear analysis [3,18,25,26,41–45]. High resolution geoarchaeological techniques have also shown to help approach single human occupation episodes [35,46–49].

A very powerful source for palimpsest dissection and for looking at isolated occupations are combustion structures. Hearths have been considered as a central element around which human activities take place [50–55] and according to ethnoarchaeological research, most activities are carried out around hearths in household areas [52–56]. Thus, combustion structures are considered as basic elements of a single occupation episode [26,36,37,57] and consequently they can aid in discerning a minimal number of occupation episodes [37].

In the Middle Paleolithic, fire residues are present in the majority of sites [58] and often comprise well-preserved hearths with distinct perimeters and internal stratigraphy. Crucially, such good preservation states could be indicative of short-term occupation or low occupation intensity, as continuous presence of humans at a site might result in obliterated raked-out hearths [6–9]. Very few Middle Paleolithic combustion structures with thick ash layers have been documented [6,8]. Most sites have yielded hearths with thin (less than 2 cm-thick) ash layers [58], suggesting a prevalence of short-lived fires, in opposition to refueled fires used over weeks and months, which have been shown to result in thick ash deposits [10–12].

According to published descriptions of Middle Paleolithic combustion structures [37,59–61], their macroscopic appearance generally conforms with the structure of simple, flat, circular, multi-layered structures exhibiting a succession of: a reddish or brownish layer at the base (here onwards Red Layer or RL), overlain by a dark brown to black layer (BL) and capped by a gray to whiteish layer (WL) [62]. Previous studies have hypothesized that the WL contains combustion-related residues, while the BL and RL are the combustion substrate representing burned topsoil beneath the fire [41,47,63]. If we consider the ephemeral archaeological fingerprint of short-term human occupations, Middle Paleolithic BL and RL deposits are unlikely to yield behavioral evidence. Instead, they hold potential to provide paleoenvironmental information because they contain residues of the natural vegetation cover prior to the occupation and the combustion event. On the other hand, any behavioral evidence associated with Neanderthal short-lived fires should be found in Middle Paleolithic WL deposits, which may conceal clues on Neanderthal behavior.

At present, although the Middle Paleolithic fire record has been widely investigated [46,64–75] it has not been sufficiently studied from a geoarchaeological perspective as to characterize the formation and post-depositional modification of individual combustion structures. This shortcoming hampers our assessment of Neanderthal occupations based on the combustion structure record as introduced in the previous paragraphs. First, identification of short-lived hearths is not possible with the naked eye because relighting, rake-out and distinguishing between single and multiple burning events requires microscopic examination [63]. Second, there are a plethora of natural taphonomic factors that may modify the macroscopic appearance of combustion structures [63]. Third, the nature of WL and BL deposits lies in their components, which are often only sedimentary and identifiable at microscopic and molecular scales. Altogether, in-depth microstratigraphic investigations of Middle Paleolithic combustion structures have the potential to provide information on Neanderthal behavior and environmental contexts and at the same time indicate isolated occupation episodes which might help to decipher the timing of short-term occupations.

To pursue such investigation, we selected stratigraphic unit X from the Middle Paleolithic site of El Salt in Spain. The wider region of El Salt displays numerous Middle Paleolithic sites: Bolomor, Abrigo de la Quebrada, Esquilleu, Castillo, Morin, Teixoneres, Axlor and Abric Romani. El Salt has yielded numerous flat combustion structures. In the field, these showed apparently good preservation states, with distinct outlines and internal stratigraphy. In certain areas and especially in stratigraphic unit X, these combustion structures are partially overlapping or superimposed. In this context, a dense archaeological palimpsest involving multiple combustion structures reflect either many successive short-term occupations with isolated combustion structures [14,17,18,26,57,76] or fewer, longer occupations with multiple synchronous ones [77–79].

Galván [24] proposed that unit X (35 cm thick, dated to 52.3 ± 4.6 Ka [80]) possibly represents recurrent, brief, low impact Neanderthal occupations, based on an integrated archaeostratigraphic study of lithic refits, raw material procurement, knapping activity, the number of single discarded flint artifacts, the minimal number of individuals obtained from zooarchaeological analyses and the number of combustion structures [25,28].

In this paper, we present the results of a geoarchaeological study coupling micromorphology and lipid biomarker analysis of a combustion structure assemblage from El Salt unit Xb to explore how these combustion structures formed. Archaeological soil micromorphology is the microscopic study of in situ preserved archaeological sediment which can aid in the characterization of depositional and postdepositional processes. Lipid biomarker analysis is the study of lipid molecular compounds that can be traced to a particular biological origin. Both methods combined will be used here to assess if the human occupations associated with the combustion structures were low impact and short-termed, as previously formulated and to provide an estimate of the time period between occupations. Given the common presence of distinct BL deposits among the unit Xb combustion structures and the fact that organic matter preservation potential in charred contexts is quite high [47,81,82], we expect to obtain significant information through the systematic microstratigraphic analysis of these combustion structure assemblages.

## 2 Material and methods

### 2.1 Site background

The Middle Paleolithic open-air site of El Salt (38°41’14”N, 0°30’32”W, 680 m a.s.l.) is located in Alcoy (Alicante, Spain) on the foot of the Mariola mountain range. It overlooks the valley of the Polop and Barchell rivers on a height of 680 m above sea level and rests against a 38 m-high Paleocene limestone wall which is covered by tufa and travertine [24,80].

Research on this site is ongoing since 1986 by a team of Universidad de La Laguna, Tenerife, Spain and involves mainly microstratigraphic and multidisciplinary site formation studies and archaeological palimpsest dissection research [24,28,47,71–73,80,83–90].

The Pleistocene sequence comprises a 6.3 m thick stratified deposit [80,84] and has been dated by thermoluminescence to between 60.7 ± 8.9 and 45.2 ± 3.4 Ka [80]. It has been divided into 13 lithostratigraphic units (XIII-I) [91], grouped into 5 different segments (Table 1) [80]. Unit X, which was dated to around 50 ka BP [80], is up to 35 cm thick and consists of archaeologically-rich brown, calcareous sandy-silty sediment [80]. It has been subdivided into two units, Xa and Xb, based on field observations of texture and color. The upper unit, Xa, is approximately 10 cm thick, is more sandy and lighter colored (brownish yellow) and unit Xb, around 10 to 14 cm thick, is more clayey and darker (brown). A recent microfaunal study proposes that unit Xb represents a more humid climate than today [83], further supported by anthracological data [71].

**Table 1:** Summary of site stratigraphy after Galván [24].

Segment	Unit	Description
**5**	I—IV	Irregular beds of gravel and cobbles in a silty clayey matrix, combines remains of late Upper Paleolithic, Epipaleolithic, Mesolithic and Neolithic remains
**4**	Upper part of V	Massive gravel in the top 20 cm and massive sandy silt, almost archaeologically sterile
**3**	Middle of V—VIII	Horizontally bedded geogenic sand and decreasing evidence of human input
**2**	IX—XII	Horizontally bedded fine sands with abundant archaeological remains and combustion residues
**1**	XIII	Archaeologically sterile horizontal carbonate platform

### 2.2 Unit Xb combustion structures

**Fig 1:**
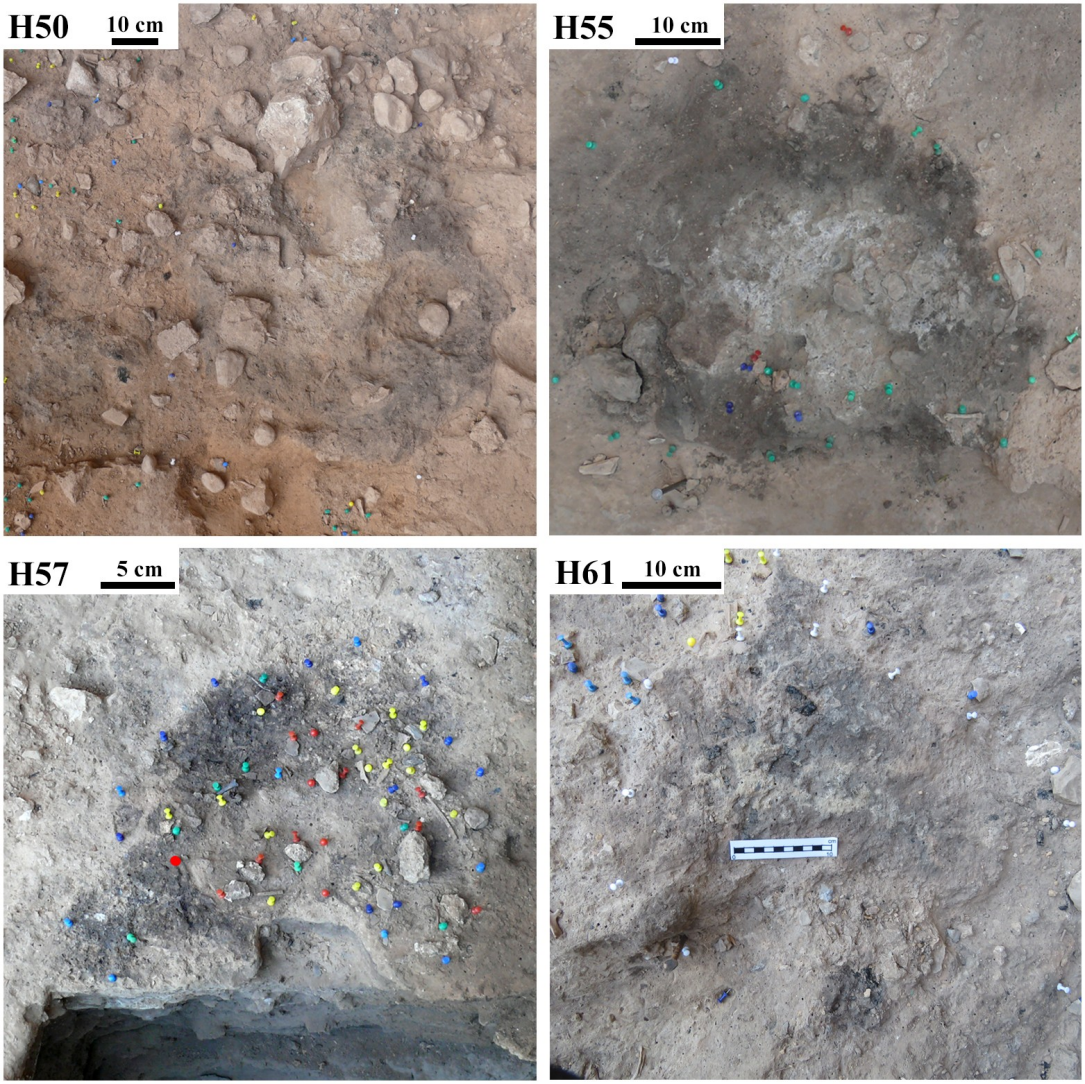
Field photos of selected combustion structures from the combustion structure assemblage.

Unit X is characterized by a high number of combustion structures concentrated near the site’s back wall: 44 in subunit Xa and 46 in subunit Xb. They are distinct flat features with variable dimensions (0.2–1.0 m in diameter) [28,47,80,90], and are stratigraphically associated with frequent faunal remains, flint flakes and anthropogenically modified cobbles [80]. The structures often overlap, yielding a combustion structure palimpsest near the back wall. In the field, the unit X combustion structures exhibit generally good preservation states, as can be observed by the presence of a distinct stratigraphic sequence (WL-BL-RL) typical of in situ flat combustion structures (Fig 1) [62]. Some of the unit X combustion structures have been characterized micromorphologically as simple, flat, open, in situ hearths whose BLs represent natural topsoils (animal excrements and plant remains in a bioturbated sedimentary matrix) [47] and some have yielded evidence of human excrements [90].

Unit Xb will be the focus of this work. Due to recent approaches in systematic sampling protocols for biomarker studies and special sample storage requirements, no biomarker samples were available from unit Xa other than those previously published in Mallol [47]. Anthracological analyses, indicate the use of dead pine wood branches as fuel and possible presence of a smoking hearth [71–73]. A microstratigraphic study combining FTIR and phytolith analyses showed relatively good preservation and mild diagenesis of the WLs, which are mainly composed of wood ash, as well as presence of wood and bark phytoliths in the BLs and WLs of the fires interpreted as ephemerally used hearths [92].

Stratigraphic unit Xb is exposed on 36 m^2^ in a total of 250 m^2^ of the site due to Holocene erosion, the old excavation from the 1960s and a preserved unexcavated part of the site [28].

### 2.3 The Xb combustion structure sample

**Fig 2:**
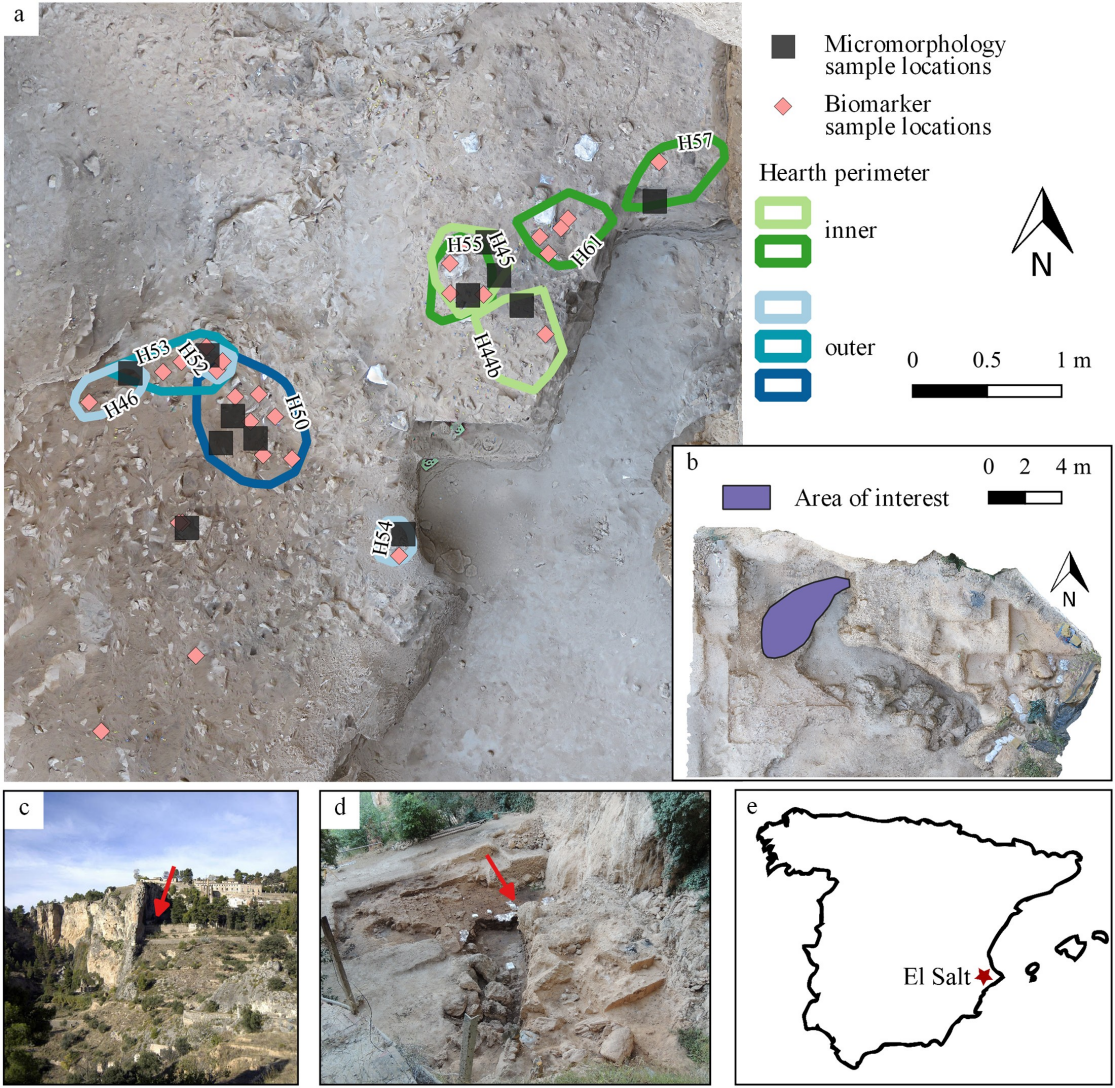
Combustion structures of unit Xb—El Salt. a) Georeferenced orthophotograph extracted from 3D photogrammetric model of the site created in the 2017 field season showing the combustion structures selected for this study (georeferenced BL perimeters) and associated micromorphology and biomarker samples. b) Georeferenced orthophotograph (same as in a) of the whole site, with indicated location of the area of interest for this study. c) Overview picture of the surrounding area of the site. Arrow indicates the position of the site. d) Overview of the site, arrow indicates the area of interest. e) Geographical location of El Salt within Spain.

This study comprises a selection of 11 subunit Xb combustion structures for which undisturbed sediment blocks and bulk sediment collected in aluminum foil were available for micromorphology and organic chemistry analysis, respectively. No permits were required for any of the samples, which are considered geological samples. Hearth H61 only yielded bulk sediment samples. This assemblage has been divided into an “inner” combustion structure cluster closer to the site’s back wall (H44b, H45, H55, H57, and H61), and an “outer” cluster located further away from the wall (H46, H52, H54, H53a, H53b, and H50) (Fig 2).

**Fig 3:**
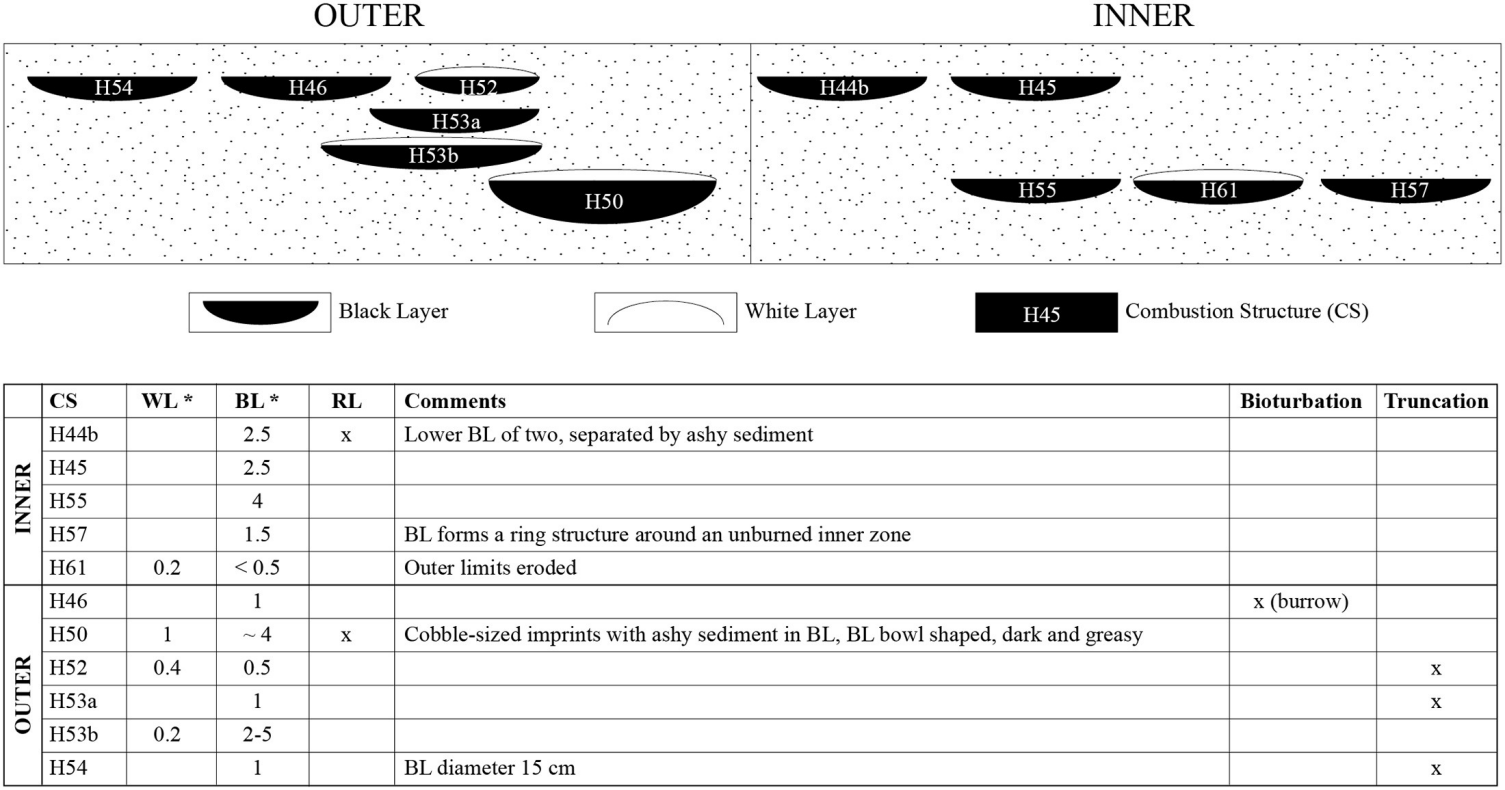
Schematic drawing showing the stratigraphic position of the selected combustion structures. Combustion structures as observed in the field with facies thickness in cm (*).

The area where the outer combustion structure cluster is found differs from the inner area in its sedimentary composition as observed in the field. In both areas, the sediment is made up of brownish yellow, massive, unsorted, gravelly silty sand with visibly abundant tufa and travertine fragments. However, in the outer area the sediment was more compact and contained frequent archaeological remains (bone fragments and flint flakes), while in the inner area the sediment was loose and stratified. Of the eleven combustion structures selected, only four showed the presence of a clear WL in the field. The rest comprised only a BL-RL succession. Nevertheless, given the presence of burnt bone and regularly circular perimeters, they are most likely to represent combustion structures. Most of the combustion features contained flint flakes, burned bone, charcoal, burnt limestone and travertine clasts. The micromorphological samples included overlying and underlying unit Xb sediment (here onwards Xb) apparently unaffected by fire. See Fig 3 for further details on the combustion features and their stratigraphic position.

### 2.4 Archaeological micromorphology and the microfacies approach

Archaeological soil micromorphology may help us to establish stratigraphic relationships, identify sedimentary and archaeological components in their context and approach depositional and post-depositional processes [93,94].

Thirteen intact, oriented blocks of sediment from the selected ten combustion structures and adjacent control sediment blocks were extracted in the field and processed into 23 petrographic thin sections (S1 Fig) in three different places. Some of the samples were processed at the Archaeological Micromorphology and Biomarker Research Lab (AMBI Lab), Universidad de La Laguna, Tenerife, Spain [95]. This involved oven drying at 60 °C for 48 hours and impregnation with a 7:3:0.1 ratio of a mixture of polyester resin (Palatal strained resin UN1866, TNK composites), styrene (Styrene monomer (CAS: 100-42-5) UN2055, TNK composites) and a catalyzer (Methyl-ethyl-ketone (Luperox, CAS: 78-93-3), TNK composites). The hardened blocks were then cut into 1 cm thick slabs using a Euro-Shatal M31100 radial saw and subsequently glued onto 9 cm x 6 cm glass slides. These were further trimmed to 1mm thickness using a Uniprec ATA Brilliant-220 precision cutting machine and ground to 30 μm thickness using a G&N MPS-RC-Geology grinding machine. For some of the samples, the 1 cm-thick resin-embedded slabs were shipped to Spectrum Petrographics Inc. (Vancouver, WA, USA) to produce 51 mm x 76 mm thin sections. Finally, some of the blocks were entirely processed by Thomas Beckmann (Schwülper-Lagesbüttel, Germany) into 9 cm x 6 cm thin sections. The thin sections were observed using a Nikon Eclipse E200 polarizing microscope with magnifications ranging between 20 and 100 and described following the standard guidelines of Stoops [96] and Nicosia and Stoops [97].

We used the concept of microfacies applied to archaeological soil micromorphology [98] as a tool to categorize our descriptions, in a similar way as presented in previous micromorphological studies [48,99–103]. This approach involves microstratigraphic determination of microfacies units (MFU) and their grouping into microfacies types (MFT) according to specific combinations of micromorphological features (recurrent certain combinations of components, porosities and microstructures) to facilitate understanding and interpretation of depositional and post-depositional processes.

### 2.5 Total, inorganic and organic carbon analysis

For testing the organic carbon content in different layers associated to combustion structures, a subset of sediment samples (WL, BL, RL, Xb, Control) were analyzed for total carbon (TC), total inorganic carbon (TIC) and total organic carbon (TOC) with a LECO SC 144DR furnace at Instituto Pirenaico de Ecología (IPE-CSIC), Spain. The information on the organic content of the WLs, BLs and RLs, as well as the control (Xb) sediment, which can help in the investigation of formation processes, is generally unavailable for Middle Paleolithic combustion features.

### 2.6 Lipid biomarkers

Biological molecular marker, or “biomarker”, is a general term applied to denote any compound used for tracing signals that help us to either reconstruct paleoenvironments or particular metabolic pathways [104]. Analysis of the molecular composition of organic compounds and their isotopic fractionation can identify specific families of plants, animals, fungi and, in the case of animals (including humans), provide information on dietary preferences. Here, we focus on lipids, which are hydrophobic, resistant compounds with relatively high preservation potential. The biomarker approach using lipids has been widely applied to archaeological materials [89,105–117] and to archaeological sediment to a lesser extent [89,118–121].

For each of the eleven combustion structures, we collected 10–15 g bulk sediment samples from their BL and 2 to 5 g bulk sediment samples from their WL. We also collected 4 stratigraphically related control samples (5–10 g) 1–2 meters away from the combustion structure area (see Fig 2).

#### 2.6.1 Lipid extraction, analysis and quantification

The bulk sediment samples were collected using sterilized metal tools, packed in aluminum foil to avoid phthalate contamination from plastic bags and stored at -20 °C to prevent bacterial degradation.

They were then processed and analyzed at the AMBI Lab. They were dried at 60 °C during 48 h, homogenized using an agate mortar and pestle and subsampled to 5 g of homogenized sediment. To obtain the total lipid extract (TLE), the sediment was mixed with 40 mL dichloromethane/methanol (DCM/MeOH) 9:1 v/v at three cycles of 30 minutes (min) in a sonicator (temperature below 30 °C) followed by centrifugation (10 min at 4700 rpm) and filtered through annealed glass wool [122]. The total extract was then evaporated with N_2_ at 40 °C, reconstituted with DCM and separated into six fractions of different polarity using solid phase extraction (SPE) on a silica gel column (1g silica 70–230 mesh and 0.1 g sand 50–70 mesh, both previously fired at 450 °C during 10 h). The elution was carried out using different solvents for each fraction (Table 2). After further evaporating with N_2_ at 30 °C, the different fractions were stored at -20 °C until measurement. Before measurement, the internal standard (IS) (8mg/L) was added and the sample was reconstituted with solvent (Table 2).

**Table 2:** Summary of the lipid fractionation procedure and reconstitution for chromatographic analysis.

Fraction No.	Fraction	Elution	Internal standard	Reconstitution volume
**1**	*n*-alkane	3/8 dead volume (DV), n-hexane	5α-androstane	150 μL of DCM
**2**	Aromatics	2 DV, 8:2 v/v n-hexane/DCM	5α-androstane	50 μL of DCM
**3**	Ketones	2 DV, DCM	5α-androstan-3-ol	50 μL of DCM
**4**	Alcohols	2 DV of 1:1 v/v DCM:Ethyl acetate (EtOAc)	5α-androstan-3-ol	40 μL of DCM + 10 μL EtOAc
**5**	Acids and diols	2 DV EtOAc	Methyl C19:0	40 μL of DCM + 10 μL EtOAc
**6**	Other compounds	2 DV MeOH	Methyl C19:0	40 μL of DCM + 10 μL EtOAc

For alcohols, trimethylsilyl (TMS) esters were obtained by adding 100 μL of N,O-Bis(trimethylsilyl)trifluoroacetamide (BSTFA) + trimethylchlorosilane (TCMS) 99:1 v/v and 100 μL of pyridine to the extract, derivatizing at 80 °C for 1 hour, drying and reconstituting with 10 μL Ethyl Acetate (EtOAc) and 40 μL DCM. Fatty acids were derivatized to methyl esters by adding 5 mL of MeOH, 400 μL of H_2_SO_4_ to the extract and heating them at 70 °C for 240 min. They were then neutralized with saturated sodium bicarbonate solution and extracted three times with 3mL hexane, dried under nitrogen and reconstituted with 40 μL DCM + 10 μl EtOAc [123].

All the different fractions were analyzed using gas chromatography (GC) with a coupled mass-selective detector (GC-Agilent 7890B, MSD Agilent 5977A) and equipped with an HP-5ms capillary column ((5% phenyl)-methylpolysiloxane, 30 m, ID: 250 μm, film thickness 0.25 μm; Agilent Technologies). The GC was programmed to an initial temperature of 70 °C (held 2 min), then heated at a heating rate of 12 °C/min to 140 °C and to a final temperature of 320 °C at a heating rate of 3 °C/min (held 15 min) with helium as carrier gas (flow of 2 mL/min). The multimode injector (MMI) was held at a split ratio of 5:1 at an initial temperature of 70 °C during 0.85 min and heated to 300 °C at a programmed rate of 720 °C/min. The MS was used under the following conditions: transfer line, ion source and quadrupole were set at 280 °C, 230 °C and 150 °C respectively, electron ionization energy level was -70 eV, and the analyzer was operated in full can mode (m/z 40–580).

Compounds were identified by comparison of their retention times and mass spectra with those of reference compounds (mix alkanes n-C8 –n-C40, 500 mg/L in DCM; 37 component FAME mix C4–C24, concentration in DCM varied from 200 to 600 mg/L; and fatty acids C26:0, C28:0, and C30:0) and comparison with the NIST mass spectra library. Quantification for *n*-alkanes was carried out using matrix-matched calibration curves [124] obtained by plotting the Area/AreaIS ratio against the concentration of reference compounds dissolved in matrix extracted with the previously described procedure. For this purpose, the four most intense fragment ions in the mass spectra were taken (m/z 43, 57, 71 and 85 for alkanes; m/z 67, 95, 81 and 245 for the IS). Correlation coefficients for calibration curves were higher than 0.9904.

Alkane concentrations are plotted in μg per gram of dry sediment (μg/gds) with their correspondent confidence intervals. To evaluate the distribution of *n*-alkanes we calculated the Carbon Preference Index (CPI), which is the ratio of the quantity of odd *n*-alkanes against the quantitiy of even *n*-alkanes, using the revised formula by Marzi et al. [125]:

$$\text{CPI}=\frac{\left(\left[\text{C}23\right]+\left[\text{C}25\right]+\left[\text{C}27\right]+\left[\text{C}29\right]+\left[\text{C}31\right]+\left[\text{C}33\right]+[\text{C}35]\right)+\left(\left[\text{C}25\right]+\left[\text{C}27\right]+\left[\text{C}29\right]+\left[\text{C}31\right]+\left[\text{C}33\right]+\left[\text{C}35\right]+[\text{C}37]\right)}{2\times \left(\left[\text{C}24\right]+\left[\text{C}26\right]+\left[\text{C}28\right]+\left[\text{C}30\right]+\left[\text{C}32\right]+\left[\text{C}34\right]+[\text{C}36]\right)}$$

Larger CPI values indicate a greater preference for odd chain lengths. CPI_area_ was calculated from the peak area of the unquantified sample, to match previous publication standards.

#### 2.6.2 Pyrolysis

Pyrolysis was carried out using a CDS Pyroprobe 500 (CDS Analytical, Oxford, PA) coupled to the Agilent GC-MS through and a split/splitless injector (AMBI Lab, La Laguna, Spain). The samples (9 mg– 13.8 mg) were placed in a quartz tube with a quartz filler rod inside. A hand-made plug of quartz wool was inserted into the tube which was then introduced into the coil of the resistively heated Pt filament. The pyrolizer was initially set at 300 °C for 1 s and then the temperature was increased to 700 °C at a rate of 20 °C/ms and held for 15 s. The transfer line was maintained at 300 °C. The GC separation and MS conditions were set at the same state as the previously used one for analysis of liquid fractions.

#### 2.6.3 Compound-specific carbon isotope analysis (CSIA) of *n*-alkanes

Compound-specific carbon isotopes of the samples were measured using a Thermo Scientific Isotope Ratio Mass Spectrometer Delta V Advantage coupled to a GC Trace 1310 through a Conflo IV interface with a temperature converter GC Isolink II (AMBI Lab, La Laguna, Spain). Samples were injected using a Programmed Temperature Vaporising (PTV) injector (splitless mode) with an evaporation step with the temperature increasing from 60 °C to 79 °C (held 30 s, rate 10 °C/min), a transfer stage increasing to 325 °C (held 3 min, rate 10 °C/s) and a cleaning temperature increased to 350 °C (held 3 min, rate 14 °C/s). A Trace Gold 5-MS (Thermo Scientific) fused silica capillary column was fitted to the GC (30 m x 0.25 mm, 0.25 μm film thickness). The carrier gas was helium at a flow rate set of 1.5 mL/min and the oven was programmed as follows: from 70 °C (held 2 min) to 140 °C at 12 °C/min, from 140 °C to 320 °C (held 15 min) at 3 °C/min. The combustion reactor temperature was maintained at 1000 °C.

Measurements were repeated three times per sample, while data was acquired and processed using Isodat 3.0 (Thermo Scientific). A *n*-alkane Schimmelmann type A6 mixture (n-C16 to n-C30) was used to normalize the δ^13^C values to the Vienna Pee Dee Belemnite (VPDB) scale. The standard deviation of those measurements was smaller than 0.50 ‰.

## 3 Results

### 3.1 Micromorphology

#### 3.1.1 Main components, porosity, microstructure and postdepositional features

**Fig 4:**
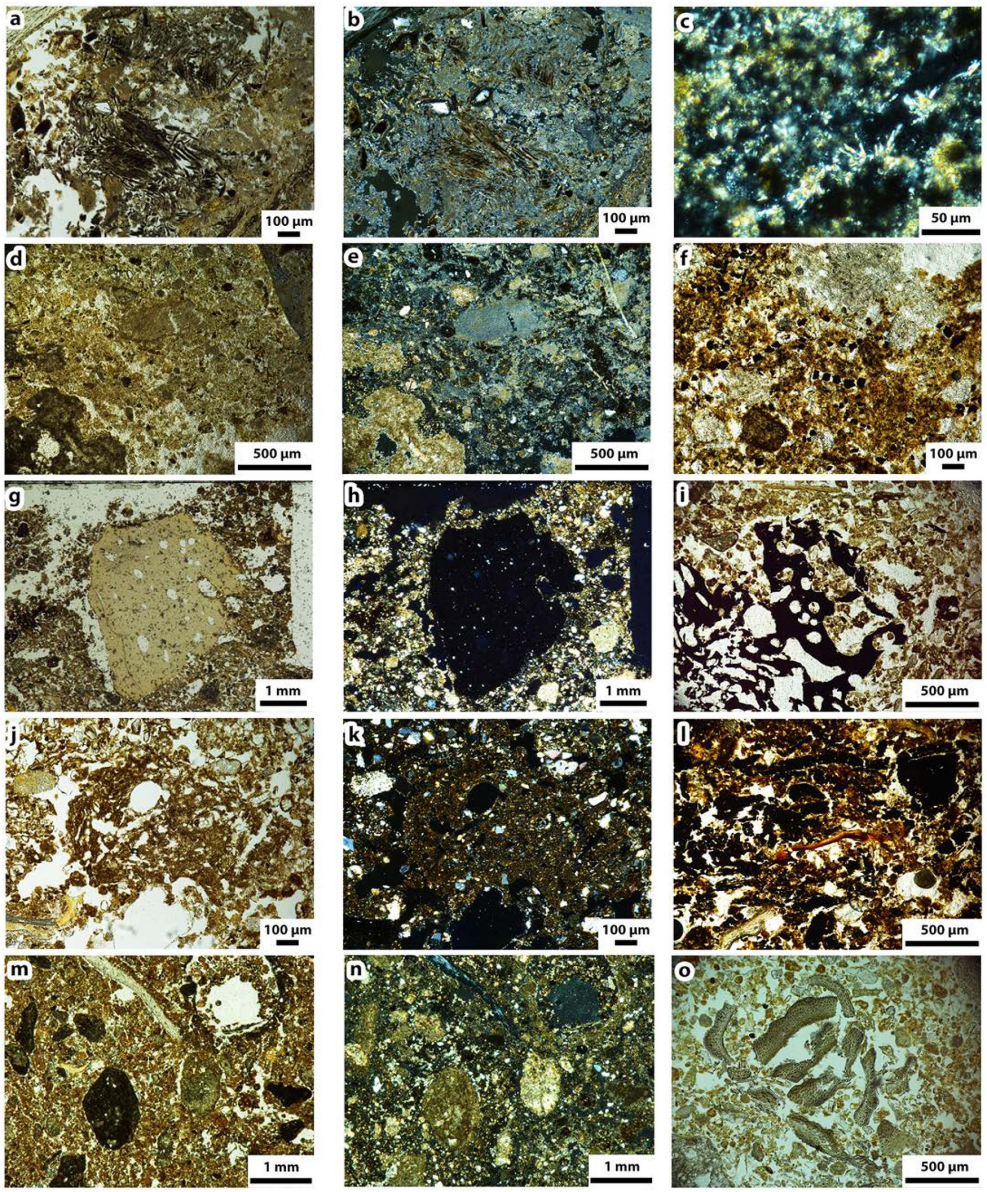
Components identified in the micromorphological samples. a) wood ash (PPL), b) wood ash (XPL), c) needle fiber calcite (XPL), d) ashes (PPL), ashes (XPL), f) unidentified square black particles (PPL), g) burnt bone (PPL), h) burnt bone (XPL), i) animal fat-derived char (PPL), j) fibrous coprolite (PPL), k) fibrous coprolite (XPL), l) blackened plant particles (PPL), m) burned tufa (PPL), n) burned tufa (XPL), o) *celtis australis* seed coats (PPL).

All the samples share a common lithological composition dominated by sand-sized limestone fragments with quartz grain inclusions, sand-sized detritic travertine and tufa fragments and fine sand-sized quartz crystals. These detritic mineral components are common or abundant in the control and Xb samples but few or rare in the BLs, where the bulk of the sedimentary mass is made up of organic components. The travertine and tufa fragments in BL and WL show signs of burning; specifically, brown or gray zones in plane polarized light (PPL) and recrystallized zones in crossed polarized light (XPL) (Fig 4m and 4n). Regarding anthropogenic and biogenic components, there are few charcoal [126] and fat derived char fragments (Fig 4i) [63], common bone [127] and tooth fragments [127], massive (Fig 4g and 4h) [128] and fibrous coprolites (Fig 4j and 4k) [129] and *Celtis sp*. seed coats (Fig 4o) [47] with variable representation and degree of burning depending on the facies. Plant residues appear browned or blackened (Fig 4f and 4l). Descriptions of these components by combustion structure and facies (WL, BL, RL) are presented in Table 3.

**Table 3:** Components identified in the combustion structures and in the sediment of Xb.

Component	Description and comments	Representative combustion structures
**Bone fragments**	Abundant in all samples, Sand-sized, predominantly small mammals, reptiles or fish and lower proportion of indistinct fragments, possibly macrofauna, in Control and RL mostly unburned, in BL commonly burned and in WL calcined	All
**Teeth**	From rodents	H46 BL, Xb
***Celtis australis* seed coats**	Locally abundant	H44b BL, H53b BL, H53b WL, H53a WL, H55 BL, Xb
**Flint flakes**	Sand-sized flakes and flake fragments	H44b BL, H50 WL H51 BL, H55 BL, Xb
**Fibrous coprolites**	Herbivore origin, abundant, in some cases, fine mass is almost entirely made up fibrous coprolites with diffuse boundaries	All
**Massive coprolites**	From omnivores or carnivores, locally frequent	Xb
**Wood charcoal**	Present in small proportions, some fragments identified as pine, rounded in BL and UL, angular in WL	H50 BL, H55 BL, H57 WL, Xb
**Blackened plant residues**	Abundant, especially in BL, fine-medium sand size, morphology suggestive of degraded plant tissue	All
**Unidentified square black particle**	Subhorizontally aligned or isolated subangular cuboids, 50 x 50 μm	H57 BL, H57 RL
**Animal fat-derived char fragments**	Very few particles, consisting of a massive black matrix with vesicular voids and fissures	Xb
**Calcitic eggshell fragments**	Common	H50 WL, Xb

**Fig 5:**
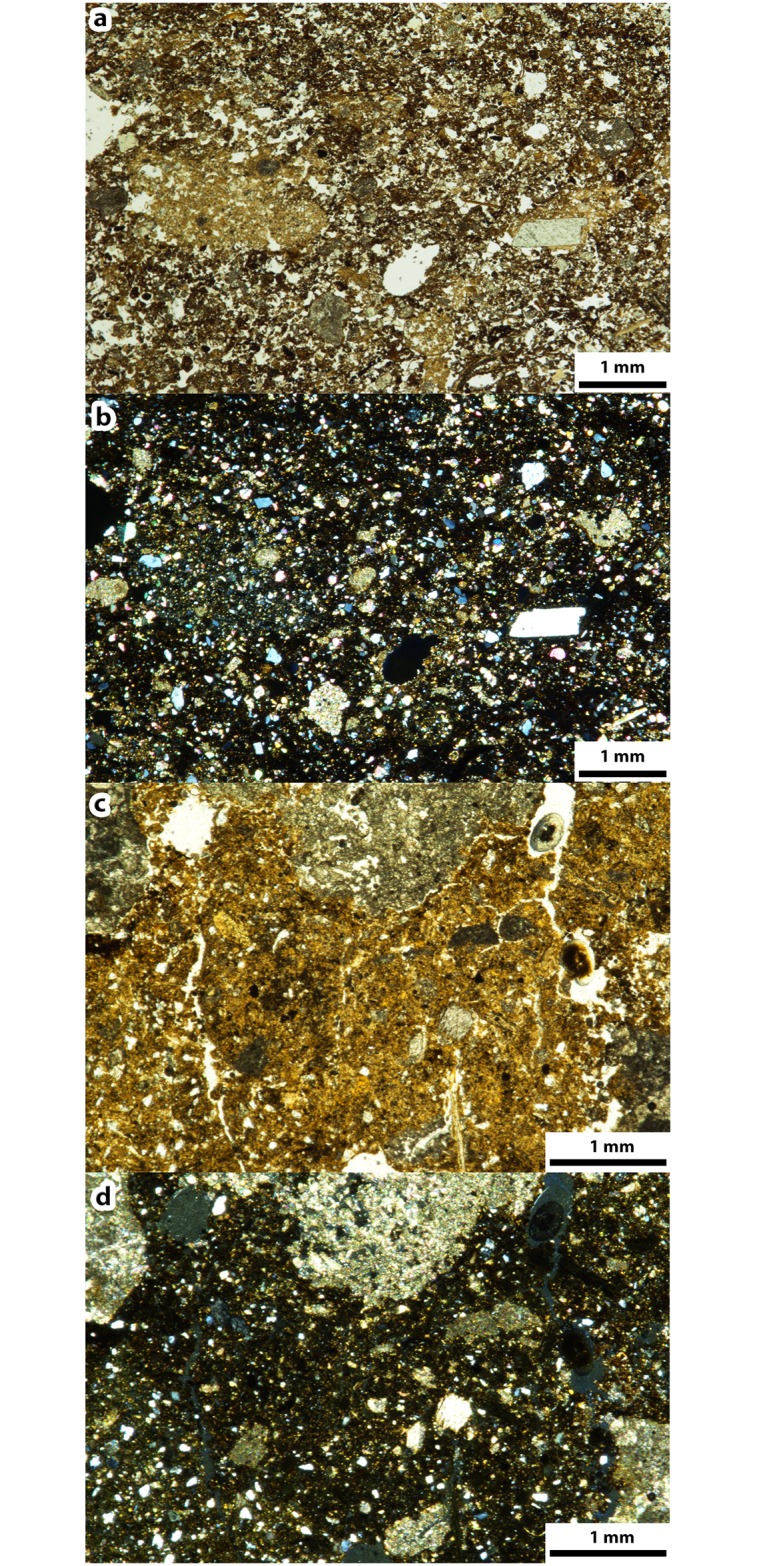
Selected matrix images. a) fibrous coprolitic matrix (PPL), b) fibrous coprolitic matrix (XPL), c) phosphatic matrix (PPL), d) phosphatic matrix (XPL).

The general fine mass is composed of undifferentiated, locally calcitic-crystallitic phosphatized clayey silt (Fig 5), in the WL it is composed of diagenetic calcitic ash (Fig 4a, 4b, 4d and 4e).

The prevailing porosity comprises vughs and compound packing voids, with localized channels and chambers and a few randomly oriented planes. The resulting microstructures are vughy, granular and intergrain microaggregate.

Mild bioturbation is the main physical postdepositional process identified. Regarding diagenesis, gypsum crystals and gypsum crystal pseudomorphs were locally observed and in all the samples the groundmass exhibits partial decalcification. The calcitic ash in the WLs was also affected by this process, exhibiting local isotropic or recrystallized domains (Fig 4c) and collapse zones with intrusive overlying sediment.

#### 3.1.2 Microfacies (MFT) types

The MFU corresponding to the observed WL, BL, RL and Xb sediment (S1 Table) were grouped into 12 microfacies types (MFT) (Table 4). These were established according to matrix color, frequency of fibrous coprolites, organic particles and degree of bioturbation. For WLs, a distinction was made according to presence/absence of wood ash and degree of diagenesis. For a detailed description of the MFT see Table 4.

**Table 4:** Microfacies types (MFT).

MFT	Layer	MFT description	Facies represented
**1**	BL	Dark brown fibrous coprolite-rich matrix with common black organic particles	H44b BL, H45 BL, H46 BL, H53b BL, H50 BL, H54 BL, H55 BL
**2**	RL	Reddish brown fibrous coprolite-rich matrix with common black organic particles	H52 RL, H53b RL, H57 RL
**3**	Xb	Weakly bioturbated brownish-yellow fibrous coprolite-rich matrix	
**4**	WL	Pale brownish-yellow fibrous coprolite-rich matrix with diagenetic wood ash	H50 WL
**5**	BL	Dark brown matrix with abundant fibrous coprolites and black organic particles	H52 BL, H53a BL, H57 BL
**6**	Xb	Brownish-yellow matrix with few fibrous coprolites and few black organic particles	
**7**	Xb	Bioturbated brownish-yellow matrix with few fibrous coprolites and few black organics	
**8**	WL	Grey matrix with in situ, well preserved wood ash	H55 WL
**9**	WL	Grey matrix of in situ, diagenetic wood ash with few calcined bone fragments	H57 WL, H52 WL
**10**	WL	Brownish-grey matrix with diagenetic wood ash	H53a WL, H53b WL

### 3.2 Total carbon

We obtained TC, TIC and TOC data on 9 samples. TC on average showed values of 6.39%, TIC values of 5.09% and TOC values of 1.30%. On average the BL yielded the highest quantity of TOC (2.08%, n = 4), followed by the control samples (TOC = 0.76%, n = 2), WLs (TOC = 0.69%) and RLs (TOC = 0.60%, n = 2). The highest percentage of TIC is shown by the BL (5.18%, n = 4) and the lowest by the Controls (4.84%, n = 2) (see Table 5).

**Table 5:** TC, TIC and TOC.

Sample	TC (%)	TIC (%)	TOC (%)
**H44b WL**	5.80	5.11	0.69
**H45 BL**	7.58	4.83	2.75
**H46 BL**	6.99	5.49	1.50
**H50 RL**	5.48	5.07	0.41
**H52 BL**	7.17	5.11	2.06
**H52 RL**	6.03	5.25	0.78
**H57 BL**	7.29	5.28	2.01
**C2**	5.30	4.42	0.88
**C4**	5.89	5.25	0.65
**Avg. WL**	5.80	5.11	0.69
**Avg. BL**	7.26	5.18	2.08
**Avg. RL**	5.75	5.16	0.60
**Avg. control**	5.60	4.84	0.76
**Avg. all**	6.39	5.09	1.30
**Min. all**	5.30	4.42	0.41
**Max. all**	7.58	5.49	2.75

### 3.3 Lipid biomarkers

#### 3.3.1 *n*-alkanes

**Fig 6:**
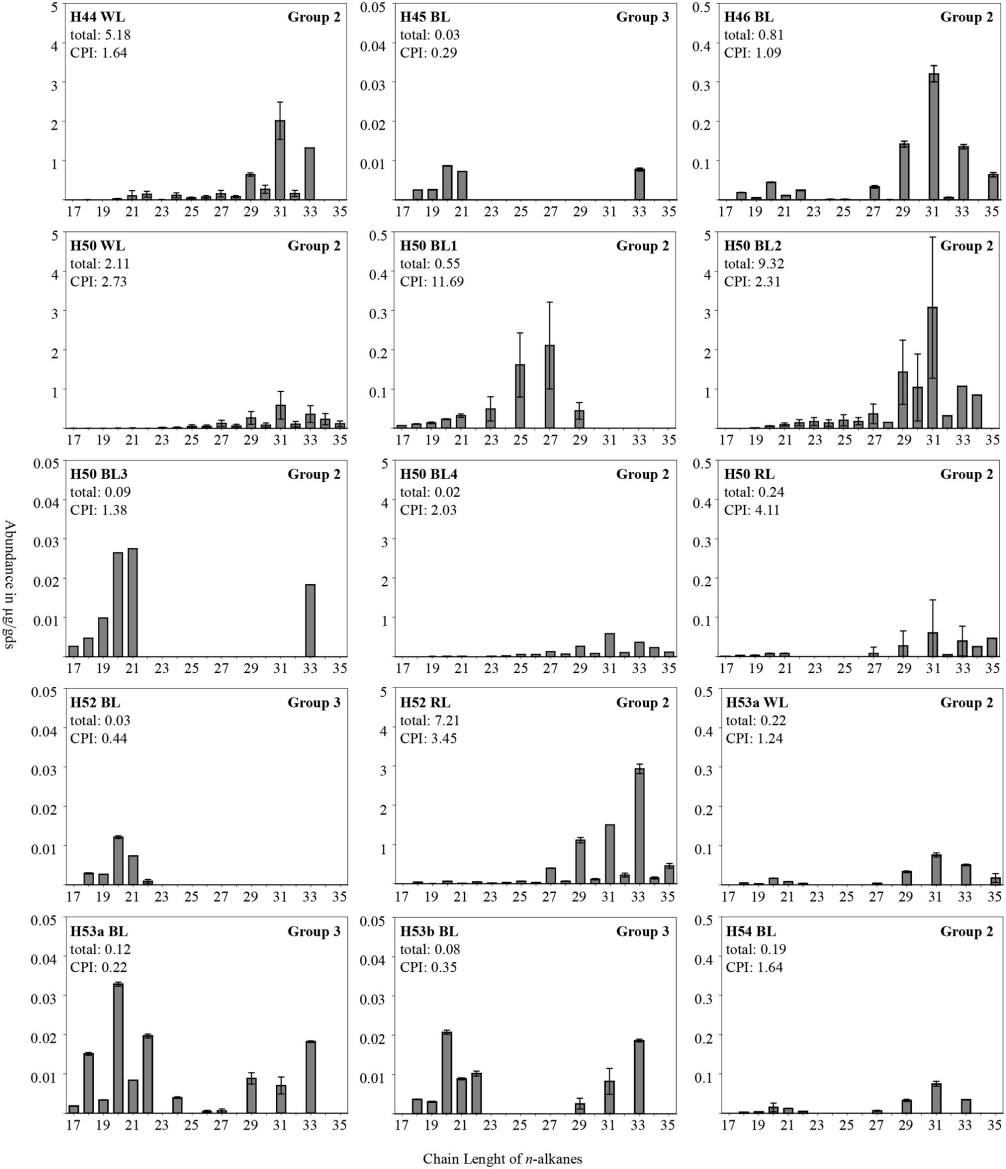
N-alkane profiles of combustion feature facies in numerical order from H44 to H54. Abundances of alkanes with chain lengths from 17 to 35 in μg/gds with three different scales on the y-axis. Error ranges are shown according to their standard deviation. For H50 BL3 and H50 BL4 the error ranges are lower than 0.004 μg/gds. Graphs show total alkane content (μg/gds), CPI and alteration group.

**Fig 7:**
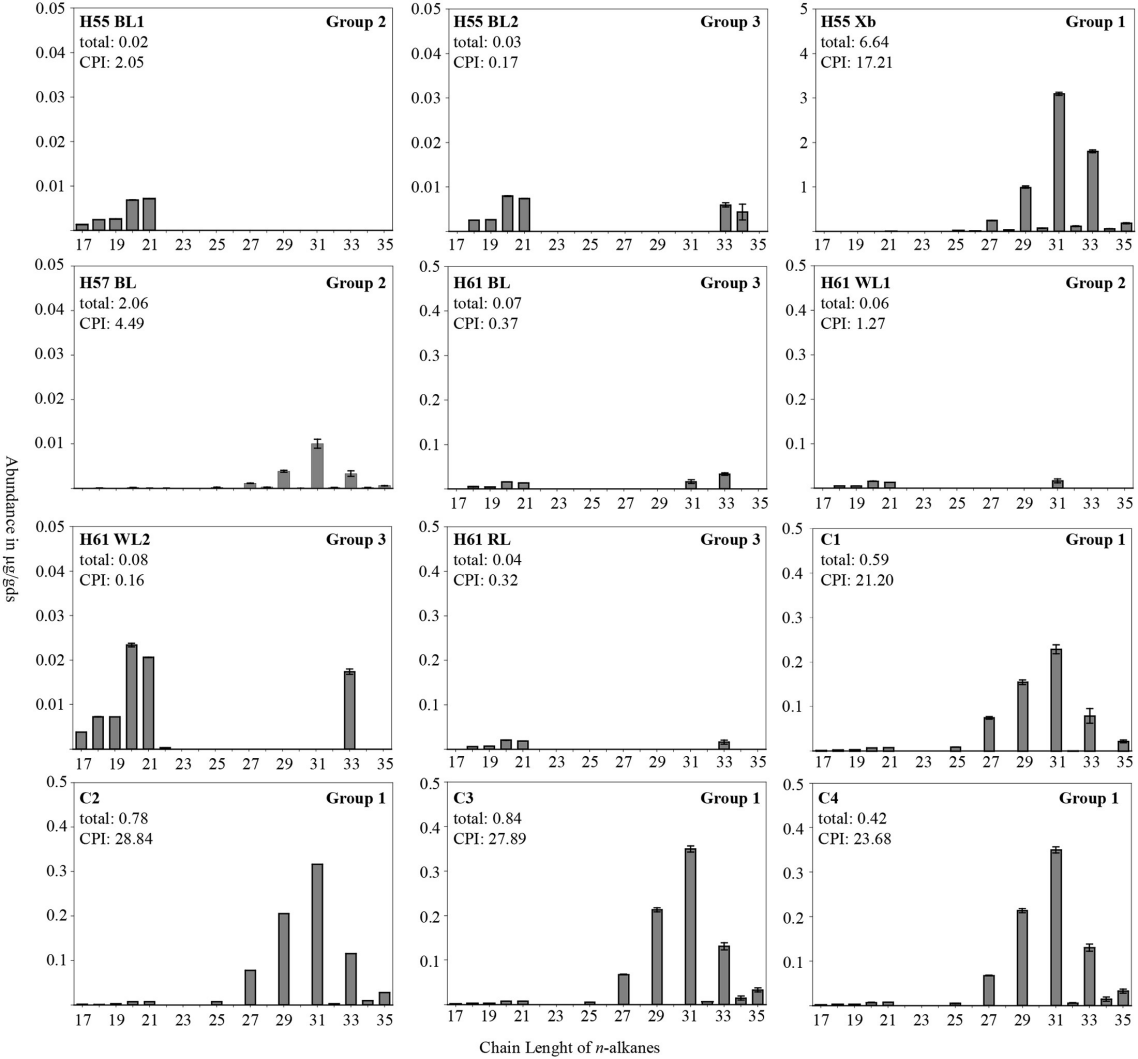
N-alkane profiles of combustion feature facies in numerical order from H55 to H61 including control samples. Abundances of alkanes with chain lengths from 17 to 35 in μg/gds with three different scales on the y-axis. Error ranges are shown according to their standard deviation. For H55 BL1 and C2 the error ranges are lower than 0.004 μg/gds. Graphs show total alkane content (μg/gds), CPI and alteration group.

We obtained *n*-alkane data from 27 loose sediment samples (Figs 6 and 7). The distribution of *n*-alkanes ranges from nC_17_ to nC_35_. The total *n*-alkane concentration within these samples varies from 0.2 μg/gds (H50 BL4) to 9.32 μg/gds (H50 BL2). Averaging the values according to their provenience results in lowest values for the BLs (n = 14) with 0.96 μg/gds, followed by WLs (n = 5) with 1.57 μg/gds, Control and Xb (n = 5) 1.85 μg/gds and RLs (n = 3) 2.50 μg/gds (Figs 6 and 7).

The CPI_area_ values (n = 27) range from 0.49 (H55 BL2) to 32.04 (CB54). Alkane profiles were divided into three groups according to their degree of alteration as indicated by the CPI values. The first group, “unaltered samples” show pronounced odd long chained peaks with a maximum at C31, none or only small even peaks and CPI_area_ values of >15. In addition to the four control samples, H55 Xb, representing Xb sediment below H55 and H50 BL1 fit into this category. Group 2 includes moderately altered samples. Their *n*-alkane profile stands out for their high, distinct long chained peaks, with the presence of short and even chained peaks. Group 2 comprises samples whose highest peaks are comparable to the unaltered samples, at C27, C31 and C33. Samples included in this category are H44b WL, H46 BL, H50 WL, H50 BL2, H50 BL3, H50 BL4, H50 RL, H53a WL, H54 BL, H55 BL1, H57 BL, H61 WL1 with CPI_area_ values ranging from 1.09 to 4.49. Group 3 represent the highly altered samples, showing a predominance in short chain and even *n*-alkanes. Samples in this category include H45 BL, H52 BL, H53a BL, H53b BL, H55 BL2, H61 WL2, H61 BL and H61 RL and their CPI_area_ lies between 0.16 and 0.44 (Figs 6 and 7).

Samples that show either no alteration (Group 1) or moderate alteration (Group 2), can be analyzed according to their dominant peaks. Most samples have C31 as dominant peak, the most dominant peak for H52 RL is C33, whereas the most dominant alkane of H50 BL1 is C27 (Figs 6 and 7).

#### 3.3.2 Other lipid fractions

Polycyclic aromatic hydrocarbons could be detected neither in the aromatic fraction nor through pyrolysis. No ketones were detected in any of the samples. However, we identified Betulin, Betulinaldehyde, Lupeol, β-Amyrin, Fucoxanthin, Stigmasta-3,5-diene, Stigmasterol, Cholesterol, 1-Hexacosanol, 1-Octacosanol, 1-Tetracosanol, Docosanol, and Squalene. The dominant fatty acids in the samples were 16:0 (palmitic acid) and 18:0 (stearic acid).

#### 3.3.3 CSIA of *n*-alkanes

**Fig 8:**
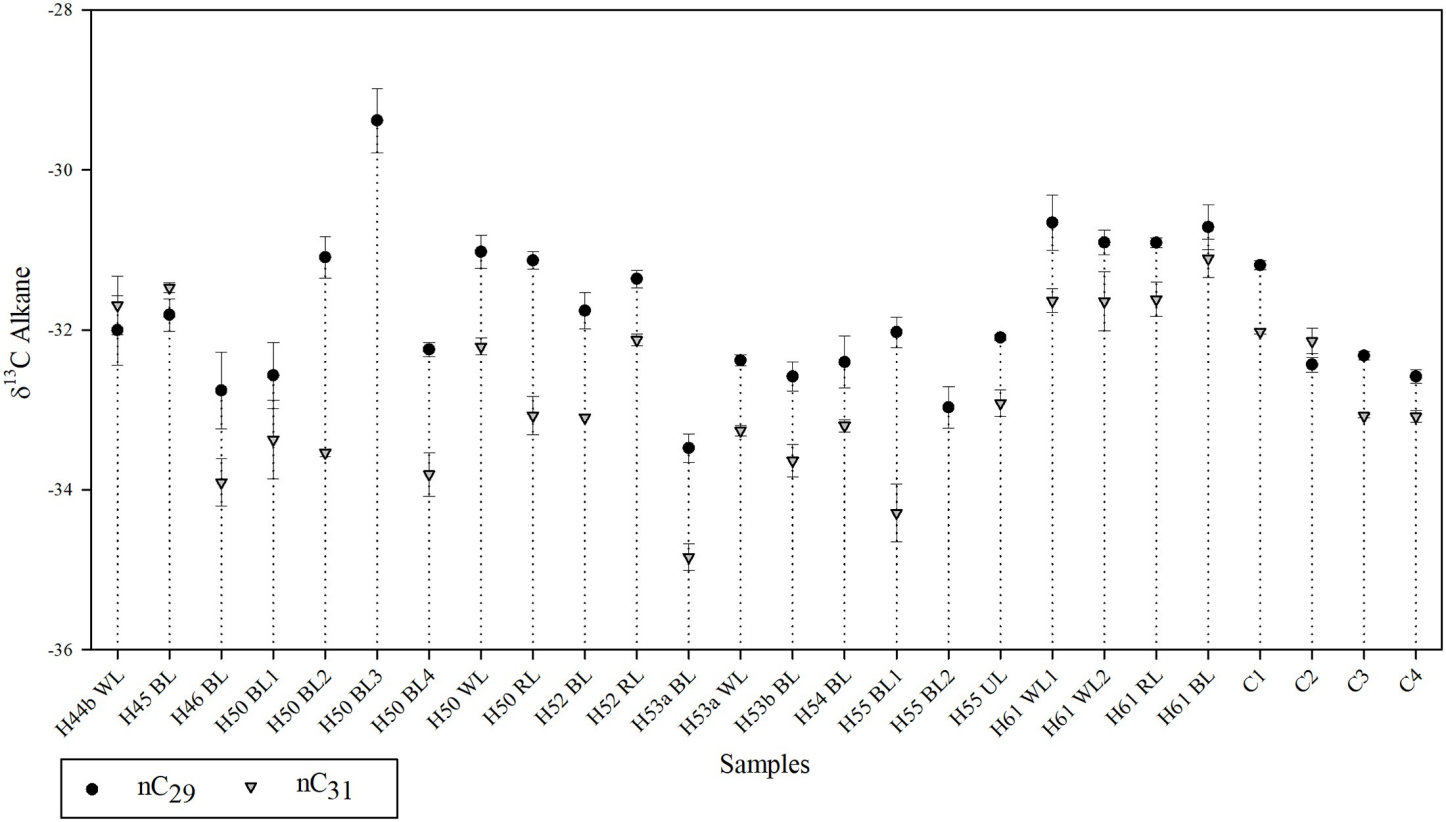
δ^13^C compound specific alkane profiles. Combustion structures on the x-axis and δ^13^C values on the y-axis, displaying the isotopic value of the alkane nC29 and nC31 with error ranges according to the standard deviation.

Compound-specific carbon isotopes of *n*-alkanes were measured for 26 samples comprising BL, WL, RL and Xb of 11 combustion structures and 4 control samples. Due to the strong predominance of odd-numbered alkanes in the samples, we focused our interpretation on the compound specific δ^13^C analysis on the long-chained, odd-numbered *n*-alkanes (C_29_ and C_31_). δ^13^C values for C_29_ range from -34.4 ‰ to -29.4 ‰, with an average of -32.0 ‰ and values for C_31_ range between -35.3 ‰ and -31.1 ‰ with an average of -32.9 ‰ (Fig 8).

## 4 Discussion

### 4.1 Formation of the unit Xb combustion structures

#### 4.1.1 Micromorphological observations

Macroscopically, the eleven combustion structures studied here are similar in size, shape, internal stratigraphy and associated archaeological material. Micromorphologically, clear ash layers could be identified in H52, H55 and H57, and diagenetically altered ash layers in H50, H53a and H53b. Four of the combustion structures showed no ash at all (H44b, H45, H46, H54). All of these combustion structures are in situ, while comprising an intact, internal stratification (RL, BL, WL), observable on the micro-scale [63]. Occasional mild bioturbation is limited to a small scale, rarely exceeding the boundaries of the individual layers. For H61 only bulk samples were available.

All the BLs represent sedimentary substrates rather than a layer of fuel residues. They mainly contain variable proportions of herbivore coprolite residues and black unidentified plant tissue.

In their composition and microstructure, the BLs of unit Xb are comparable to those from the overlying unit Xa [47]. They consist of a loose, fibrous coprolite-rich sedimentary matrix with common geogenic detritus, microfaunal bone remains, degraded topsoil plant matter, a few charcoal fragments and very few flint fragments. Fibrous coprolites and fibrous coprolite-rich sediment have been previously reported in herbivore dung deposits [129,130]. Thus, the BLs possibly represent undisturbed natural topsoils occupied by herbivores and later charred by anthropogenic fire. Crucially, the presence of common geogenic detrital mineral grains and scattered, finely comminuted degraded plant matter and microfaunal bone indicates they are natural topsoils and not purely dung, nor fuel. The plant matter is present in the form of unidentified fibrous or massive black particles originating from topsoil vegetation [131]. The original morphology of the perishable plant parts (parenchyma or collenchyma) was not observed, therefore the plant matter must have undergone a certain degree of degradation prior to combustion, possibly corresponding to amorphous lignin-rich tissue [132,133] or remains of the epidermis, which are hard to decompose [134,135]. The unit Xb control sediment samples yielded very few, scattered black particles. The longest sequence of hearths in the studied area consists out of four roughly overlapping combustion structures (H52, H53a, H53b and H50). Considering the geogenic and pedogenic nature of their respective BLs the site must have been unoccupied by humans in between each of the combustion events.

Human impact was not observed apart from the effect of burning that resulted in the BLs. Only rare flint fragments and macrofaunal bone remains were identified and no signs of trampling were observed. Charcoal fragments are also scarce. Some of them have been identified as conifer, possibly *Pinus nigra or Pinus sylvestris*, in accordance with previous anthracological evidence from unit Xb [71]. Given the detrital, mixed composition of the BLs and the roundness and smoothness of the charcoal fragments which are few, these are possibly reworked, deriving from nearby, preceding combustion events.

Regarding the WLs, no anthropogenic components were identified in any of them, suggesting that the activities carried out around them did not involve tossing refuse into the fire, at least in significant amounts, nor cooking practices involving deposition of bone or char residues in the fire. Most of them showed signs of postdepositional reworking or diagenetic alteration. Strong bioturbation and mixing with the overlying sediment were observed in both H50 WL and H53 WL, in addition to strong decalcification in H53 WL.

#### 4.1.2 Total organic carbon

The preservation of organic matter (TOC) in BLs is better than in WLs, RLs and Xb. This agrees with previous studies by Mallol et al. [47] underlining the potential of charred organic matter to preserve biomarker fingerprints. This implies that temperatures in the BLs are sufficiently low as to preserve organic matter but at the same time high enough to produce non-biodegradable combustion residues.

#### 4.1.3 Lipid biomarkers

The lipid compounds identified conform with the BL deposits representing former topsoils. The triterpenoids betulin, lupeol and β-amyrin are angiosperm biomarkers [136–138]. Terpenoids originate mainly from leaves, bark [138] and resins [138,139] while triterpenoids are indicative of angiosperms and diterpenoids of gymnosperms [140]. In unit Xb, the anthracological record indicates that conifers represent the predominant wood fuel type [71–73] and thus diterpenoids originating from tree resin were expected to be present in the combustion structure sediment [138,141,142], especially since conifers, as members of the Pinaceae family, produce high quantities of resin [139]. However, no gymnosperm biomarkers were identified in any of the BL samples. This agrees with the BLs representing a charred vegetated surface under the fires and not fuel residues. Considering that terpenoids would mainly derive from leaves of trees in the immediate surroundings of the site and only triterpenoids are present, we can assume that the site was surrounded by angiosperms prior to the combustion events. Although diterpenoids are naturally underrepresented because they are less abundant than triterpenoids [140], an absolute absence of diterpenoids makes it less likely that gymnosperms grew in the vicinity of the site. Consequently, the conifer wood used to fuel the fires, was possibly collected off-site.

Other indicators of angiosperm input are aliphatic compounds including 1-Hexacosanol, 1-Octacosanol, 1-Tetracosanol, and Docosanol [136], all of which are present in our samples. A prevalence of nC_29_ and nC_31_ as dominant *n*-alkanes points to the presence of leaves from higher plants as well [122,143–146]. A possible source of leaves could be the Celtis tree (*Celtis australis*) since Celtis seed coats are present throughout the thin sections, likewise, Celtis phytoliths have been previously reported for unit Xa [47,92].

Given the interpretation of BLs as topsoil, it is important to determine whether the black microscopic plant residues are blackened by charring, biodegradation or both. Our micromorphological observations suggest some degree of biodegradation (see above). However, our biomarker data show the presence of stigmasta-3.4—diene and stigmasterol which are biomarkers of burnt plant biomass [136]. In addition, no homophanes or hopenes, which are bacterially derived triterpenes [137] were identified. We only observed a few black plant parts in the unit Xb sediment control samples and the *n*-alkane profiles of these samples do not show any alteration on the molecular scale of the *n*-alkanes. Thus, bacterial degradation is unlikely, and the varying degrees of alteration in the *n*-alkane profile observed in most of our BLs and WLs is possibly due to burning and are comparable to previously reported data from plant biomass burning [147]. This raises the question of the temperatures reached in the BLs as a result of subsurface heat penetration.

Given that our samples show a predominant angiosperm leafy content with a strong probability of the presence of Celtis trees, we can compare our data to a recent study involving controlled heating experiments on *Celtis australis* leaves, bark, branches and twigs [122] to provide estimates of the temperatures at which our BLs were heated. Accordingly, our strongly altered samples (Group 3) were possibly burned to temperatures higher than 350 °C [122] but possibly lower than 500 °C [148], moderately altered samples (Group 2) were heated in the range between 150 °C and 350 °C [122] and unaltered samples (Group 1) were not heated to more than 150 °C [122]. Overall, these temperatures are within the range documented for experimental hearth substrates with an organic-rich substrate [47,149]. Other experimental fires conducted on plain substrate or organic poor substrates reach higher subsurface temperatures ([150] and references therein).

One exception is BL1 of H50, which showed dominant peaks in nC_25_ and nC_27_ suggesting the presence of twigs or branches [122]. Jambrina-Enríquez et al. [122] show that this *n*-alkane profile changes with combustion at temperatures higher than 150 °C, suggesting that the H50 BL did not reach temperatures higher than 150° C and thus the twigs or branches were not fuel but topsoil residues.

Regarding our biomarker data for the WLs, none of them shows highly altered *n*-alkane profiles, contrary to our expectations. This lack of alteration might be explained by the effect of bioturbation and diagenesis, which is prominent in these samples as shown by our micromorphological data. Possibly, the *n*-alkane profile obtained contains a mixed signal of both the original ash layer and an unburned sediment postdating the hearths. Despite this mixing, the CPI values of the WLs indicate the lowest alkane preservation, whereas the best alkane preservation according to the CPI was recorded in the control samples. This suggests that even when mixing WL and Xb the CPI remains lower than that of the BL. Further work is required to better understand the effects of intrusive ashy sediments on CPI values.

#### 4.1.4 Compound-specific stable carbon isotopes of *n*-alkanes

The δ^13^C values of nC_29_ and nC_31_ alkanes range between -35.3 ‰ and-29.4 ‰. These values are indicative of plants with a C3 photosynthetic pathway [122,151–153]. Important to note is that the isotopic values fluctuate up to 5 ‰ in the sample set. This variation is not related to the combustion structures or their facies (WL, BL, RL). In fact, the highest variability was observed in different samples within the BLs of H50. This variability could be explained by differences in plant functional types (e.g. grasses, deciduous angiosperms, evergreen gymnosperms) [154]. As previously discussed, the *n*-alkane profile of H50 BL1 stands out for showing the presence of twigs and branches, suggesting a more heterogeneous composition with regards to plant matter. Additionally, the micromorphological data from H50 BL, which shows a pronounced fibrous planar microstructure, demonstrates a distinctive pattern exclusive to H50 BL.

Since most of the samples belong to combustion contexts, the question arises as to the effect of heating on isotopic values. Above 330–360 °C an increasing depletion in ^12^C takes place, which can be attributed to the cleavage of 12C-12C bonds [155]. On the other hand, Diefendorf et al. [156] report a shift of compound-specific *n*-alkane δ^13^C values up to 2 ‰ in either direction depending on species and chain length, at a temperature higher than 200–250 °C. Thus, a shift in the compound-specific *n*-alkane δ^13^C values would corroborate that BL and WL represent in fact burning if all other parameters are ruled out. As shown by our *n*-alkane data, a good number of our samples appear to have been heated to more than 250 °C. Even so, the heterogeneous composition of the organic matter (different plant tissues) should be considered. Jambrina-Enríquez et al. [122] reported that the xylem from Celtis branches and twigs was enriched by ∼2 ‰ in ^13^C relative to leaves, whereas bark was depleted by ∼3 ‰ in ^13^C relative to leaves and that shifts of δ^13^C values with increasing combustion temperature are different within plant tissues (leaves, bark and xylem). Therefore, our isotope data must be taken with caution and further basic research comparing compound-specific carbon isotope values of fresh and burned plants is needed.

### 4.2 Implications for the timing and intensity of Neanderthal occupations at El Salt Unit Xb

The good preservation states of the unit Xb combustion structures as observed in the field are corroborated by our joint micromorphological and geochemical data. The degree of bioturbation as well as diagenesis is moderately low, and the BLs represent in situ combustion substrates. These combustion substrates were natural vegetated surfaces with decayed leaves and herbivore excrements, which possibly accumulated when humans were away. No signs of human activity were observed besides the lighting of simple, flat fires on these surfaces. Material remains observed microscopically are very few burnt bone residues in the ash layers (WLs) and trace amounts of scattered bone and flint flakes in BLs, RLs and Xb. Only ephemeral signs of human activity were found in the WLs microscopically. On this basis and focusing on the relative stratigraphic position of the four superimposed, diachronic combustion features from the outer area (H50, H53b, H53a, H52), we propose that the unit Xb combustion structure assemblage represents at least 4 low-impact human occupation episodes (minimal number of occupations) separated by unknown periods of time during which the place was not occupied by humans. No stratigraphic discontinuities, signs of erosion or long time pedogenesis was observed, suggesting that the amount of time of site abandonment was limited.

The charred plant matter contained in the BLs may provide additional clues to narrow down the timing of human occupations at El Salt unit Xb. Aside from blackening and possible shrinkage [126] charring is not expected to change the original morphology of plant tissue. Thus, we can assume that the morphology of the black particles we observed reflect the state of the plant matter present in the topsoil at the time of burning. As previously discussed, our microscopic charred plant matter, morphologically amorphous lignin-rich tissue, possibly reflects remains of moderately degraded angiosperm plants. Given this evidence, we can hypothesize that the fires were not made in the fall, in which case they would have charred a fresh leaf cover. Generally, the brittle parts of leaves (parenchyma and collenchyma) decay within a few months after deposition [132], so human occupations in El Salt unit Xb possibly did not take place during the fall season.

Furthermore, our results also suggest that the Neanderthal groups that occupied El Salt during the formation of unit Xb did not stay there for prolonged amounts of time, which would have resulted in obliterated, re-lit hearths and higher amounts of anthropogenic remains. Instead, they arrived, occupied the place leaving the previously uninhabited natural surfaces intact, except for localized burning, and left before these had been obliterated by natural or anthropogenic processes.

### 4.3 Inter- and Intra-site comparison of the hearth assemblage

To corroborate our interpretation of brief human occupations separated by relatively long periods of time and further explore consequences of short-term occupations at El Salt, we need to compare our data with other stratigraphic units or sites in the Iberian Peninsula comprising combustion structure, lithic and faunal assemblages.

El Salt stratigraphic unit Xa has been rigorously analyzed using different methods. Micromorphology conducted on selected combustion structures also yielded BLs representing charred plant litter [47]. The black layers are up to 2 centimeters thick and some are covered with a thin WL [47]. Alkane data from those combustion structures appear to support our data in having the highest peaks at 29 and 31 and showing signs of heat alteration [47]. This evidence also points to relatively long periods of site abandonment in unit Xa.

Lithic raw materials of unit Xa were gathered within a 20 km radius and mostly within a 5 km radius [25,157] supporting short occupation episodes and high mobility [1,13]. Flint cores are uncommon and are possibly part of the habitually transported gear which together with evidence of tool recycling was interpreted as reflecting high group mobility [25]. The low sedimentation proposed in unit Xa that made tool recycling possible is supported in the nature of the palimpsest of unit Xb, whereas the approximately 2 cm thick organic soil covering the site at arrival might have made a straightforward recovery of tools more challenging. Indirect evidence of carnivore activity like marks from teeth and digestion, isolated anatomical remains and coprolites were detected [25,87,158], supporting long periods of site abandonment and short-term occupations [1,16,21,159]. Finally, a layer of up to 7 cm-thick of archaeologically sterile sediment was recorded within unit Xa, interpreted as a period of site abandonment of decades to more than a century [25]. Lithic and faunal analyses of unit Xb are ongoing and preliminary observations point to similar animal processing, lithic raw material procurement and technological patterns as observed for Xa.

Other Middle Paleolithic Iberian sites dating roughly to the same period as unit Xb have also yielded rich anthropogenic combustion records, but these cannot be directly compared to our dataset because in most cases there is no associated micromorphological or lipid biomarker data. Available field descriptions of combustion structures show the presence of BLs mainly in open air or rock shelter settings [27,74,160], with similar shape and thickness to those in our dataset and could also represent short term combustion events [27,160]. In caves combustion structures are composed of a reddened substrate in connection with burned anthropogenic material and occasionally ashes [161–164], with a lack of BLs formed from charring organic-rich substrate, as expected for a sheltered space unaffected by soil forming processes. Micromorphological analyses have been conducted at Bolomor cave where hearths were identified as having a short duration [161,162], and Esquilleu cave, which is at a considerable distance from El Salt where hearths exhibit trampling and diagenesis [46,165]. A comparison with the unit Xb structures might be confounding, as those fires were made in cave settings, whereas our dataset is from an open-air setting. Accordingly, micromorphological comparison with our dataset is not possible.

A third site rich in combustion remains, similar age and associated micromorphological data is Abric Romaní. Abric Romaní is a rock shelter site around 370 km north of El Salt. Combustion structures exhibit BLs composed of plant and organo-mineral remains, with humified, charred or calcined components in level J [166] and level O [74]. Combustion structures in both levels are considered to be identical [74]. Even though conifer wood was used to fuel the fires, the carbonaceous polymorphs present in the BLs are not derived from the fuel [74]. Comparing our data to microphotographs of combustion structures of Abric Romaní we observe similarities between their graphitic vitreous carbon and our amorphous plant material [74]. Thus, fires in Abric Romaní, like the ones in El Salt, were possibly made on organic rich substrates after relatively long abandonment periods.

## 5 Concluding remarks

Our study of El Salt unit Xb adds to the few Middle Paleolithic sites in Europe that have yielded well-preserved combustion structures as confirmed through microstratigraphic analyses: Abric Romaní [57,74,75], Gorham’s Cave [167], Grotte XIV [168,169], Klissoura Cave [170], Lakonis Cave [171], Pech de l’Azé IV [172,173], Roc de Marsal [174], Vanguard Cave [167], and El Salt unit Xa [86]. The BLs of combustion features from El Salt unit Xb were formed through anthropogenic burning of an underlying organic-rich natural surface in an environment vegetated by angiosperms, including Celtis trees and intermittently occupied by herbivores and Neanderthals. These fires were fueled by wood gathered away from the site. The paucity of Neanderthal occupation during the formation of El Salt unit Xb, possibly representing at least four successive low impact human occupation events, with enough time in between to allow for the formation of an organic rich soil and occupations most likely in winter, spring and/or summer.

The results from this study show the good preservation of lipid molecules in Paleolithic sedimentary contexts, supporting the implementation of organic chemistry in geoarchaeological research. In the case of combustion features, our data show not only that lipid molecules preserve well in burnt sediment but also how lipid biomarker analysis may contribute basic information to corroborate evidence of burning, provide temperature estimates and aid in the taxonomic identification of charred organic matter, all of which are a necessary complement to micromorphological data. Our biomarker results were limited by a lack of reference material on lipid biomarkers and compound-specific carbon isotopes of plants, plant functional types and seeds burned at different temperatures.

In sum, micromorphology combined with lipid biomarker analysis is a powerful approach to investigate anthropogenic combustion-related archaeological contexts from a microstratigraphic perspective which can contribute valuable information on the timing and intensity of Neanderthal occupations as well as the natural setting of the site. All these are key factors of group mobility and settlement patterns.

## Supporting information

S1 Fig Thin sections of the sample set with indication on the position of the combustion structure. Sample set divided into inner area and outer area according to the provenience of the samples (see Fig 2).
(TIF)

S1 Table Micromorphological description of MFU with accompanying MFT. (PDF)
