# Supplementary figures and images for: Insights into the timing, intensity and natural setting of Neanderthal occupation from the geoarchaeological study of combustion structures: A micromorphological and biomarker investigation of El Salt, unit Xb, Alcoy, Spain
Source: PLoS One. 2019 Apr 24;14(4):e0214955. doi: 10.1371/journal.pone.0214955 (PMC6481795; doi:10.1371/journal.pone.0214955)

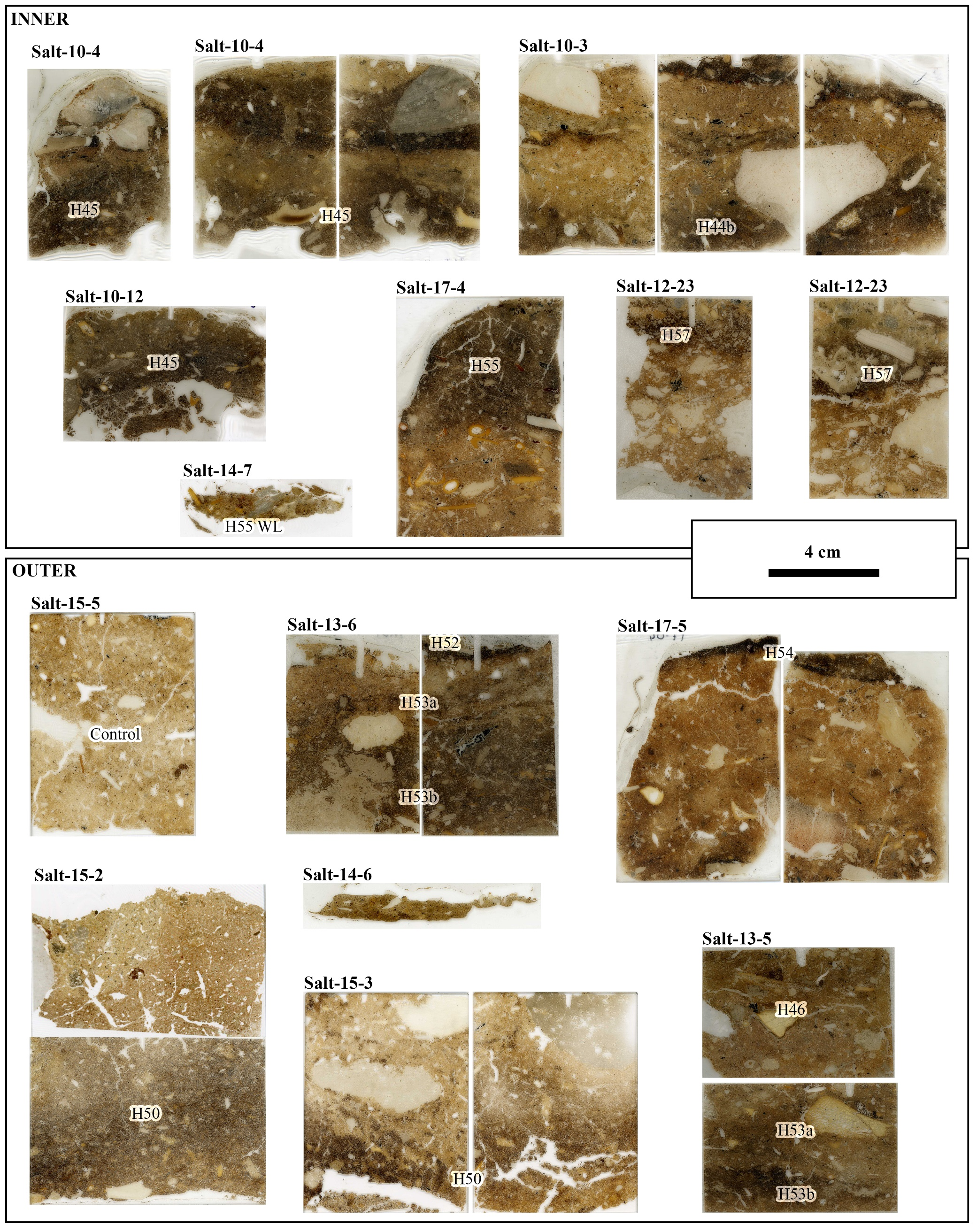

Supplement: S1 Fig — Sample set divided into inner area and outer area according to the provenience of the samples (see Fig 2). (TIF) [file pone.0214955.s001.tif]
