# Supplementary material for: Insights into the timing, intensity and natural setting of Neanderthal occupation from the geoarchaeological study of combustion structures: A micromorphological and biomarker investigation of El Salt, unit Xb, Alcoy, Spain
Source: PLoS One. 2019 Apr 24;14(4):e0214955. doi: 10.1371/journal.pone.0214955 (PMC6481795; doi:10.1371/journal.pone.0214955)
Supplement: S1 Table — (PDF) [file pone.0214955.s002.pdf]

MFU/ID	Voids	Microstructure and porosity	Lithology class	Components	Post depositional processes	MFT
Salt-10-3-MFU-1<br>H44b - BL	complex packing voids (2),<br>vughs (1),<br>channels (1),<br>planes (1)	weakly developed granules,<br>intergrain microaggregate microstructure,<br>double spaced porphyric c/f $_{20\mu m}$ 1/3	L 4	mostly burned bone (1S),<br>charcoal (1S),<br>other carbonized plant (1S),<br>animal fat derived char (1S),<br>undetermined carbonized particles (1S),<br>fissured flint (1S),<br>fibrous coprolite (2S),<br>celtis (2S)	bioturbation,<br>passage feature	1
Salt-10-3-MFU-2<br>Xb	vughs (2),<br>channels (1)	massive pedality,<br>vughy microstructure,<br>open porphyric c/f $_{20\mu m}$ 1/4	L 4	unburnt bone (1S),<br>other carbonized plant (1S),<br>fibrous coprolite (1S),<br>celtis (2S)	bioturbation	3
Salt-10-4-MFU-1<br>H45 - BL	planes (1),<br>channels (1),<br>compound packing voids (1),<br>vughs (2)	no pedality,<br>intergrain microaggregate,<br>double spaced porphyric c/f $_{20\mu m}$ 1/3	L3	burned bone (2S),<br>fibrous coprolites (1S),<br>other carbonized plant (2S),<br>celtis (1S)		1
Salt-10-4-MFU-2<br>Xb	channels (1),<br>compound packing voids (1),<br>vughs (2)	no pedality,<br>intergrain microaggregate,<br>double spaced porphyric c/f $_{20\mu m}$ 1/3	L1	celtis (2S),<br>unburned bone (1S),<br>fibrous coprolite (1S)		6
Salt-10-12-MFU-1<br>H45 - BL	vughs (2),<br>channels (1)	no pedality,<br>vughy microstructure,<br>single spaced porphyric c/f $_{20\mu m}$ 1/2	L3	tooth (1S),<br>burned bone (1S),<br>celtis (1S),<br>fibrous coprolite (1S)		1
Salt-12-23-MFU-1<br>Xb	planes (2),<br>vesicles (1),<br>channels (1),<br>chambers (1)	weakly developed S blocky peds,<br>S blocky microstructure,<br>double spaced porphyric c/f $_{20\mu m}$ 1/2	L1	unburnt bone (2S),<br>burned bone (1S),<br>rotten charcoal (1S),<br>fibrous coprolite with celtis (1S),<br>square black particle (see picture) (1S),<br>massive black particle (1S),<br>fibrous coprolite with celtis (1S),<br>celtis (1S)	bioturbation<br>(passage feature,<br>infillings from layer above)	7
Salt-12-23-MFU-2<br>H57 - RL	vughs (1),<br>complex packing voids (1)	weakly developed granules,<br>vughy-granular microstructure,<br>open porphyric/f $_{20\mu m}$ 1/5	L1	orange unburnt bone (1S),<br>burned bone (2S),<br>square black particle, linear, horizontal (3A),<br>celits (1S)		2
Salt-12-23-MFU-3<br>H57 - BL	planes (2),<br>channels (1),<br>vughs (2)	moderately developed S blocky peds,<br>complex microstructure,<br>single spaced porphyric c/f $_{20\mu m}$ 1/3	L2	burnt bone (2S),<br>fibrous long chains of black material without pores (1S),<br>square black particles (3A)		5
Salt-12-23-MFU-4<br>H57 - WL	vughs (2)	weakly developed peds,<br>vughy microstructure,<br>close porphyric c/f $_{20\mu m}$ 1/3	L 3	calcined bone (3S),<br>burned bone (1S),<br>square black particles (1S),<br>charcoal (1S),<br>other carbonized plant (1S),<br>fibrous coprolite, some burned (1S),<br>massive coprolite (1S)		9
Salt-13-05-MFU-1	vughs (1),<br>channels (2),<br>chambers (3)	weakly developed granules,<br>chamber microstructure,<br>open porphyric c/f $_{20\mu m}$ 1/5	L 4	unburned bone (1S),<br>undetermined carbonized particle (1R),<br>fibrous coprolite with undefined borders , weathered (1S)	bioturbation	3

Salt-13-05-MFU-2<br>H53b - BL	vughs (2),<br>vesicles (1)	no pedality<br>vughy microstructure,<br>open porphyric c/f $_{20µm}$ 1/4	L 5	unburned bone (2S),<br>fish bone, some burned (2R),<br>burnt bone (1S),<br>rotten charcoal (1S),<br>other carbonized plant (2S)		1
Salt-13-05-MFU-3	vughs (1),<br>vesicles (3),<br>channels (1),<br>chambers (1)	no pedality<br>vesicular microstructure,<br>open porphyric c/f $_{20µm}$ 1/5	L1	unburnt bone (1S),<br>burnt bone (1S),<br>undetermined carbonized particle (1S)	needle fiber calcite,<br>bioturbation	3
Salt-13-05-MFU-4<br>H53a - BL	vughs (2),<br>vesicles (2),<br>channels (1),<br>chambers (1)	no pedality<br>vughy, vesicular microstructure,<br>open porphyric c/f $_{20µm}$ 1/5	L1	burnt bone (1S),<br>undetermined carbonized particle (1S)	water percolation	5
Salt-13-05-MFU-5	vughs (2),<br>vesicles (1),<br>channels (1),<br>chambers (1)	no pedality,<br>vughy microstructure,<br>no related distribution c/f $_{20µm}$ 1/3	L1	unburnt bone (2S),<br>fish bone, some burned (1S),<br>undetermined carbonized particle (1S),<br>flint (1A)	bioturbation	3
Salt-13-05-MFU-6<br>H46 - BL	vughs (2),<br>vesicles (1),<br>channels (1),<br>chambers (1)	no pedality,<br>vughy, vesicular microstructure,<br>open porphyric c/f $_{20µm}$ 1/4	L1	burnt bone (1S),<br>unburnt bone (1S),<br>tooth (1A),<br>other carbonized plant (1S)		1
Salt-13-05-MFU-7<br>Xb	vughs (2),<br>vesicles (1),<br>channels (1),<br>chambers (1)	no pedality<br>vughy, vesicular microstructure,<br>open porphyric c/f $_{20µm}$ 1/7	L1	unburnt bone (3S),<br>burned bone (1S),<br>animal fat derived char (1S)	bioturbation	3
Salt-13-06-MFU-01<br>Xb	channels (2),<br>vughs (2)	no pedality<br>vughy microstructure,<br>open porphyric c/f $_{20µm}$ 1/6	L1	unburnt bone (2S),<br>fissured charcoal (1R),<br>burned/dark fibrous coprolite (1S)	bioturbation	3
Salt-13-06-MFU-02<br>H53b - RL	vughs (2)	no pedality<br>vughy microstructure,<br>open porphyric c/f $_{20µm}$ 1/4	L1	unburnt bone (3S),<br>burned fish bone (1R)<br>fissured charcoal (1R),<br>other carbonized plant (1R)	gypsum crystal (1A)	2
Salt-13-06-MFU-03<br>H53b - BL	vughs (2),<br>channels (1),<br>planes (2)	no pedality<br>vughy microstructure,<br>open porphyric c/f $_{20µm}$ 1/4	L1	unburnt bone (1S),<br>rotten charcoal (1S),<br>other carbonized plant (1S),<br>fibrous burned coprolite (1S),<br>celtis (2S)		1
Salt-13-06-MFU-04<br>H53b - WL	vughs (3),<br>channels (1)	no pedality<br>vughy microstructure,<br>close porphyric c/f $_{20µm}$ 1/2	L 4	burnt bone (2S),<br>other carbonized plant (1S),<br>burned celtis (1R)		10
Salt-13-06-MFU-05<br>H53a - BL	vughs (2),<br>planes (1)	no pedality<br>vughy microstructure,<br>close porphyric c/f $_{20µm}$ 1/4	L 4	burnt bone (1S),<br>undetermined carbonized particle (1S)		5
Salt-13-06-MFU-06<br>H53a - WL	vughs (2),<br>vesicles (1)	no pedality<br>vughy microstructure,<br>open porphyric c/f $_{20µm}$ 1/5	L 4	mostly unburned bone (1S),<br>celtis (some burned, 1S),<br>humified organic material (1S)		10

Salt-13-06-MFU-07<br>H53a - BL	channels (2),<br>planes (1)	no pedality,<br>channel microstructure,<br>open porphyric, $c/f_{20\mu m}$ 1/5	L 4	burned bone (1S),<br>other carbonized plant (1S)	bioturbation	5
Salt-13-06-MFU-08<br>H53a - WL	planes (1),<br>channels (1),<br>chambers (2),<br>vughs (1)	weakly developed plates,<br>channel microstructure,<br>open porphyric $c/f_{20\mu m}$ 1/4	L 4	calcined bone (1S),<br>unburned bone (1S)	bioturbation	10
Salt-13-06-MFU-09<br>H52 - RL	planes (1)	weakly developed plates,<br>platy microstructure,<br>open porphyric $c/f_{20\mu m}$ 1/3	L 4	bone (1S)		2
Salt-13-06-MFU-10<br>H52 - BL	planes (2)	weakly developed plates,<br>platy microstructure,<br>open porphyric $c/f_{20\mu m}$ 1/3	L 5	burned bone (1S),<br>other carbonized plant (2S)		5
Salt-13-06-MFU-11<br>H52 - WL	vughs (3)	no pedality,<br>vughy microstructure,<br>open porphyric $c/f_{20\mu m}$ 1/5	L1	unburned bone (1S),<br>fibrous coprolite (3S)		9
Salt-14-6-MFU-1<br>H50 - WL	compound packing voids (2)	moderately developed crumbs,<br>crumb microstructure,<br>enaulic $c/f_{20\mu m}$ 1/2	L 4	unburned bone (1S),<br>undetermined carbonized particle (1S),<br>flint mainly horizontally oriented (3A),<br>fibrous coprolite (1S),<br>shell (1S)		4
Salt-15-2-MFU-1<br>Xb	vughs (2),<br>channels (1)	massive peds,<br>spongy, vughy microstructure,<br>open porphyric $c/f_{20\mu m}$ 1/3	L 4	burned bone (1S),<br>unburnt bone (2S),<br>other carbonized plant (1S),<br>animal fat derived char (1S),<br>undetermined carbonized particles (1S),<br>flint (1R),<br>fibrous coprolite (1S),<br>weathered celtis (1S),<br>shells (1S)	bioturbation	3
Salt-15-2-MFU-2<br>H50 - BL	planes (1),<br>vughs (2),<br>vesicles (1),<br>channels (1)	massive peds,<br>vughy microstructure,<br>open porphyric $c/f_{20\mu m}$ 1/3	L2	mostly burned (2S),<br>rotten, fissured charcoal (1S),<br>other carbonized plant (2S),<br>fragmented accumulated particles (2S),<br>undetermined carbonized particle (2S),<br>fibrous coprolite, some burned (2S)		1
Salt-15-2-MFU-3<br>Xb	channels (1),<br>vughs (2),<br>chambers (1),<br>vesicles (1)	massive peds,<br>vughy, channel microstructure,<br>open porphyric $c/f_{20\mu m}$ 1/4	L2	unburned bone (1S),<br>burned bone (1S),<br>undetermined carbonized particle (1S),<br>massive coprolite (1S),<br>celtis vertically oriented (1S)	bioturbation	3
Salt-15-2-MFU-4<br>H50 - WL	channels (2),<br>complex packing voids (1),<br>vughs (1)	weakly developed crumbs,<br>crumb microstructure,<br>no related distribution $c/f_{20\mu m}$ 1/4	L 6	unburnt bone (1S),<br>celtis vertically oriented (2S),<br>fibrous calcified mosses (1S)	bioturbation	4

Salt-15-3-MFU-1<br>Xb	vughs (2),<br>planes (1),<br>channels (1)	massive peds,<br>vughy microstructure,<br>open porphyric c/f $_{20\mu m}$ 1/4	L 4	apparently burned bone (1S),<br>charcoal (1S),<br>other carbonized plant (1S),<br>undetermined carbonized particle (1S),<br>massive coprolite with cracks (1S, fissured),<br>fibrous coprolite incorporated in matrix (1S)	bioturbation	3
Salt-15-3-MFU-2<br>H50 - BL	vughs (2),<br>planes (1),<br>channels (1)	massive peds,<br>vughy microstructure,<br>open porphyric c/f $_{20\mu m}$ 1/3	L 4	apparently burned (2S),<br>fissured charcoal (1S),<br>other carbonized plant (1S),<br>undetermined carbonized particle (1S),<br>fibrous coprolite incorporated in matrix (1S)		1
Salt-15-3-MFU-3<br>Xb	vughs (1),<br>channels (1),<br>chambers (1),<br>complex packing voids (1)	weakly developed S blocky peds,<br>granules,<br>channel, vughy microstructure,<br>loose spaced porphyric c/f $_{20\mu m}$ 1/2	L1	unburnt bone (2S),<br>burnt bone (1S),<br>undetermined carbonized particle (1S),<br>celtis (2S)	bioturbation	3
Salt-15-5-MFU-1<br>Xb	vughs (2),<br>chambers (1)	massive peds,<br>vughy microstructure,<br>open porphyric c/f $_{20\mu m}$ 1/3	L1	unburnt bone (1S),<br>charcoal (1S),<br>other carbonized plant (1S),<br>celtis (1S),<br>massive coprolite (1S)	dark staining around chambers,<br>bioturbation	7
Salt-15-5-MFU-2<br>Xb	vughs (2),<br>chambers (1),<br>channels (1)	massive peds,<br>vughy microstructure,<br>open porphyric c/f $_{20\mu m}$ 1/2	L1	unburnt bone (2S),<br>calcined bone (1R),<br>charcoal (1S),<br>other carbonized plant (1S),<br>fibrous coprolite (1S),<br>silicified celtis (1S),<br>massive coprolite (1S)	bioturbation (infilling)	7
Salt-15-5-MFU-3<br>Xb	channels (2),<br>chambers (1),<br>planes (1),<br>vughs (1)	no pedality<br>channel microstructure,<br>open porphyric c/f $_{20\mu m}$ 1/2	L1	unburnt bone (2S),<br>calcined bone (1S),<br>charcoal (1S),<br>other carbonized plant (1S),<br>fibrous coprolite (1S),<br>massive coprolite (1S)	dark staining around channel,<br>phosphates,<br>bioturbation	7
Salt-15-5-MFU-4<br>Xb	vughs (2),<br>channels (2)	no pedality<br>vughy microstructure,<br>open porphyric c/f $_{20\mu m}$ 1/3	L1	unburnt bone (2S),<br>charcoal (1S),<br>other carbonized plant (1S),<br>silicified celtis (1S)	dark staining around channel,<br>bioturbation	7
Salt-15-5-MFU-5<br>Xb	vughs (2),<br>channels (2)	no pedality<br>vughy microstructure,<br>open porphyric c/f $_{20\mu m}$ 1/2	L1	unburnt bone (2S),<br>burned bone (1S),<br>charcoal (2S),<br>animal fat derived char (2S),<br>fibrous coprolite (1S),<br>massive coprolite (2S)	dark staining around channel,<br>bioturbation	7
Salt-17-4-MFU-1<br>Xb	channels (2),<br>vughs (3)	no pedality,<br>vughy microstructure,<br>double spaced porphyric c/f $_{20\mu m}$ 1/3	L1	unburned bone (3S),<br>celtis (2S),<br>other carbonized plant (1S),<br>fibrous coprolite (1S)	gypsum pseudomorph (1A),<br>bioturbation	7

Salt-17-4-MFU-2<br>H55 - BL	vughs (2),<br>channels (2)	no pedality,<br>channel microstructure,<br>open porphyric, c/f $_{20\mu m}$ 1/5	L3	burned bone (2S),<br>charcoal (1R),<br>celtis (1S),<br>other carbonized plant (1S),<br>flint (1S),<br>massive coprolite (1R)	bioturbation	1
Salt-17-4-MFU-3<br>H55 - WL	vughs (2)	no pedality,<br>vughy microstructure,<br>single spaced porphyric c/f $_{20\mu m}$ 1/2	L3	ash (3),<br>burned bone (2S),<br>calcined bone (1S)		8
Salt-17-5-MFU-1<br>Xb	channels (1),<br>planes (2),<br>vughs (2),	no pedality,<br>intergrain microaggregate,<br>open porphyric c/f $_{20\mu m}$, 1/5	L1	bone unburned (3S),<br>bone burned (1S),<br>celtis (2S),<br>teeth (1S),<br>other carbonized plant (1S)	gypsum pseudomorph (2A)	6
Salt-17-5-MFU-2<br>H54 - BL	vughs (1)	massive pedality,<br>massive microstructure,<br>single spaced porphyric c/f $_{20\mu m}$ 1/1	L3	celtis (2S),<br>burned bone (1S),<br>other carbonized plant (3S)		1

Lithology Class	
L1	Limestone, vcs-fs (2S),<br>Quartz, s-vfs (2S),<br>Tufa, fg-cs (2S),<br>Travertine, mg-vcs (1S)
L2	Limestone, cs-ms (1S)<br>Quartz, s-vfs (1S)<br>Tufa, vfg-cs (1S) Travertine, cs (1S)
L3	burned Limestone, cs-ms (1S)<br>Quartz, s-vfs (1S)<br>burned Tufa, vfg-cs (1S)<br>burned Travertine, fg-cs (1S)
L4	Limestone, ms-fs (2S)<br>Quartz, s-vfs (2S)<br>Tufa, cs (1S)
L5	Limestone, ms-fs (1S)<br>Quartz, s-vfs (1S)<br>Tufa, cs (1S)
L6	burned Limestone, cs-ms (1S)<br>Quartz, s-vfs (1S)<br>burned Tufa, vfg-cs (1S)<br>burned Travertine, cs (1S)

Abbreviations

S: subrounded/subangular
R: rounded
A: angular

1: rare to few
2: common to frequent
3: abundant

fg: fine gravel
vfg: very fine gravel
vcs: very coarse sand
cs: coarse sand
ms: medium sand
fs: fine sand
vfs: very fine sand
s: silt
c: clay
